# Global Psoriasis Burden 1990–2021: Evolving Patterns and Socio-Demographic Correlates in the Global Burden of Disease 2021 Update

**DOI:** 10.3390/healthcare13192437

**Published:** 2025-09-26

**Authors:** Deng Li, Siqi Fan, Jiayi Song, Haochen Zhao, Linfen Guo, Peiyu Li, Xuewen Xu

**Affiliations:** 1 Department of Plastic and Burns Surgery, West China Hospital, Sichuan University, Chengdu 610207, China; lidengdelight@gmail.com (D.L.); 2022151620431@stu.scu.edu.cn (S.F.); 2024224025140@stu.scu.edu.cn (J.S.); 2022224020085@stu.scu.edu.cn (L.G.); 2023224025399@stu.scu.edu.cn (P.L.); 2 Department of Urology, West China Hospital, Sichuan University, Chengdu 610207, China; 2017141241080@stu.scu.edu.cn

**Keywords:** psoriasis, age-standardized rates, incidence, prevalence, global burden of disease

## Abstract

**Background:** Psoriasis is a chronic immune-mediated disease affecting approximately 43 million individuals worldwide. While previous studies provide certain insights, there remains different conclusions and a lack of a comprehensive analysis regarding the burden of psoriasis. In response to ongoing therapeutic advances and a growing patient population, this study utilizes the Global Burden of Disease (GBD) 2021 estimates to characterize the spatiotemporal evolution of the psoriasis burden from 1990 through 2021. By integrating these biological, geographic, and socioeconomic determinants, this study aims to inform more targeted and effective health policy planning. **Methods:** To track changes over time, the Estimated Annual Percentage Change (EAPC) was determined using a linear regression model. In addition, a frontier analysis was utilized to investigate the link between psoriasis burden and socio-demographic progress. Furthermore, geographically weighted regression was used for the spatial econometric assessment of EAPC, age-standardized rates (ASRs), and Human Development Index (HDI) covariance structures across nation-states. **Results:** Between 1990 and 2021, the global burden of psoriasis increased consistently, with ASRs exhibiting a positive correlation with the Socio-demographic Index (SDI). High-SDI regions reported the highest burden, while high–middle-SDI regions experienced the steepest rise. **Conclusions:** This study reveals an increasing global psoriasis burden (1990–2021) through systematic analyses, indicating distinct regional progression patterns. These findings advocate for geographically tailored strategies to alleviate healthcare system pressures.

## 1. Introduction

Psoriasis is an immune-mediated inflammatory disorder that predominantly presents as erythematous, scaly plaques on the skin [1]. While the characteristic erythematosquamous plaques are diagnostic hallmarks, the disease spectrum also extends to destructive arthropathy in 17.58% of cases and cardiometabolic comorbidities that amplify mortality risks [2,3,4,5,6]. The visible nature of skin lesions, especially when affecting socially sensitive areas such as the scalp, face, and genitalia, results in profound psychosocial consequences. Surveys reveal that up to 20% of psoriasis patients in Western countries experience depression [7,8,9,10,11].

Psoriasis demonstrates significant epidemiological variation worldwide, across different regions and populations. This variation derives from multiple mediators including environmental impacts, climate, health-related behaviors, genetic predispositions, and socio-demographic progress [12,13]. Global analyses reveal striking geographical gradients, with age-standardized prevalence rates that vary 14-fold between low-prevalence regions like East Asia (0.14%) and high-prevalence regions like Australasia (1.99%) [14]. Despite this, the epidemiology of psoriasis suffers from critical data limitations, particularly concerning incidence and detailed demographic segmentation. Currently, 76% of countries lack data on psoriasis epidemiology [15]. In low- and middle-income countries, the reliance on small-scale studies leads to uncertain prevalence estimates. Many studies also lack data disaggregated by sex or precise age categories, hindering the generation of age-standardized estimates and obscuring our understanding of the demographic impact.

The Global Psoriasis Atlas (GPA) has recently conducted the most comprehensive mapping to date of psoriasis epidemiology, compiling incidence and prevalence data across more than 100 countries [15]. Despite its significant contributions, the GPA’ s geographic coverage remains incomplete, and the reported estimates exhibit considerable variability due to inconsistencies in data collection, case definitions, and reporting standards. These limitations underscore the continued need for globally harmonized, model-driven estimation strategies.

To assess the population-level impact of psoriasis, it is essential that we employ robust metrics. One such metric is Disability-Adjusted Life Years (DALYs), which integrate the years lost due to premature mortality and productive life compromised by disability. Each DALY reflects one year of optimal health forfeited, collectively illustrating the gap between current health status and an ideal, disease-free state. Socioeconomic development levels significantly influence this burden through healthcare access inequalities, effectively captured by the Socio-demographic Index (SDI) and Human Development Index (HDI). SDI, a composite measure of income per capita, educational attainment, and fertility rates, helps predict a health system’s capacity to mitigate DALYs. Similarly, HDI provides complementary insights into the societal infrastructure supporting chronic disease management through factors such as life expectancy, education, and gross national income per capita.

We used the Global Burden of Disease (GBD) 2021 database to assess the global burden of psoriasis by generating age-, sex-, and location-specific DALY estimates, and examine key socioeconomic determinants. To identify regionally coherent trends, we applied hierarchical clustering to national EAPCs in incidence, prevalence, and DALYs. For a more nuanced analysis of socioeconomic influences, we integrated the SDI with components of the HDI. To quantify the disparities in the disease burden relative to development levels, we constructed SDI–ASR frontier curves to estimate the efficiency gaps. We further decomposed DALYs by age, sex, and location to enhance the spatiotemporal resolution, and developed future burden projections under alternative development scenarios. These methodological innovations, underpinned by the comprehensive GBD dataset, enable a more granular and spatially explicit characterization of the global psoriasis burden, thereby informing precision surveillance and context-specific policy interventions.

## 2. Methods

### 2.1. Overview

The GBD 2021 dataset provides comprehensive insights into disease patterns, epidemiological trends, and the burden of disease worldwide. It synthesizes data across 204 countries and territories and employs standardized methodologies that have been extensively documented in earlier publications [16]. GBD 2021, released in 2023 by the Institute for Health Metrics and Evaluation (IHME), reflects the most recent finalized estimates available at the time of release and includes data from 1990 through 2021.

### 2.2. Study Data

This evaluation is based on information drawn from the GBD database, which has offered thorough global health evaluations since beginning in 1991 [16,17,18]. With every update, the GBD has broadened its range, including additional causes, risk factors, and geographic areas, alongside enhanced age-specific analyses. For the 2021 GBD release, data were collected from 328,938 distinct sources, resulting in over 60.7 billion estimates. These figures span 25 different age groups, starting from birth up to those aged 95 and older, across 204 countries and territories [19]. The world is segmented into 21 geographies and 7 broader regions, offering separate estimates for males, females, and the general population. A global team of collaborators executed thorough data reviews and analysis [20].

The processes of data retrieval and selection adhered to the GATHER (Guidelines for Accurate and Transparent Health Estimates Reporting) principles [21]. To quantify the burden of the disease, DALYs were utilized to quantify health-adjusted life expectancy reductions attributable to morbidity and premature mortality. Annual figures on psoriasis incidence, prevalence, DALYs, the age-standardized incidence rate (ASIR), age-standardized prevalence rate (ASPR), and age-standardized DALY rate (ASDR) were gathered for the period from 1990 to 2021 via the Global Health Data Exchange (GHDx). The GBD 2021 dataset synthesizes epidemiological information from a wide array of sources, including population-based surveys, disease registries, hospital records, insurance claims, and peer-reviewed literature. To ensure comparability across countries, the GBD employs a standardized Bayesian meta-regression modeling framework (DisMod-MR 2.1), which enforces a consistent case definition and adjusts for variations in data quality, availability, and structure. This approach allows for internally consistent and globally comparable estimates of disease burden, including DALYs.

### 2.3. Statistical Analyses

Firstly, to investigate the global burden trends of psoriasis over the period from 1990 to 2021, the calculation of the Estimated Annual Percentage Change (EAPC) was pivotal. The use of EAPC has been thoroughly reviewed in existing literature [22]. Briefly, utilizing longitudinal modeling, temporal fluctuations in age-standardized rate (ASR) trajectories were quantified via regression-based analytical methods. Secondly, a hierarchical clustering analysis grouped the EAPCs concerning incidence, prevalence, and DALYs, thereby identifying GBD regions sharing analogous EAPC characteristics. Furthermore, examining the link between the burden of psoriasis and the advancements in socio-demographic factors was achieved through frontier analysis. Here, the effective difference—measured as the discrepancy between the actual DALYs of a nation and its frontier—highlights the untapped health potential inherent to that nation’s developmental situation. Relationships among EAPCs, ASRs for years 1990 and 2021, and the HDI across various countries were also scrutinized. Additionally, data spanning 1990–2021 from the GBD database facilitated the examination of the relationship between SDI and ASRs, with SDI values assigned between 0 (minimum) and 1 (maximum). The detailed study workflow, including data retrieval, preprocessing, modeling, and statistical analyses, is summarized in the methodological flow chart (Supplementary Figure S1).

Statistical analyses were performed using R v3.5.3, with significance defined as *p* < 0.05 [23]. Estimates were derived from 1000 sample iterations, reporting 95% uncertainty intervals (UI).

## 3. Results

### 3.1. Global Burden Attributable to Psoriasis by Age and Sex

In 2021, the global number of prevalent psoriasis cases reached 43.0 million (95% UI: 41.7–44.3), increasing from 23.1 million (95% UI: 22.3–23.8) in 1990 (an 86.43% increase over three decades), with ASPR increasing from 477.7 (95% UI: 462.1–492.7) to 515.95 (95% UI: 500.2–531.6) per 100,000 person-years. Parallel to these trends, the incidence reached 5.1 million new cases (95% UI: 5.0–5.3), accompanied by an ASIR rise from 57.0 (95% UI: 55.3–58.8) to 62.0 (95% UI: 60.1–63.9) per 100,000. Moreover, the total DALYs escalated from 2.0 million (95% UI: 1.4–2.7) to 3.7 million (95% UI: 2.7–4.9), accounting for approximately 9% of all skin disease-related DALYs. The corresponding ASDR increased from 41.1 (95% UI: 29.8–54.9) to 44.4 (95% UI: 32.2–59.2) per 100,000 (Table 1).

**Figure 1:**
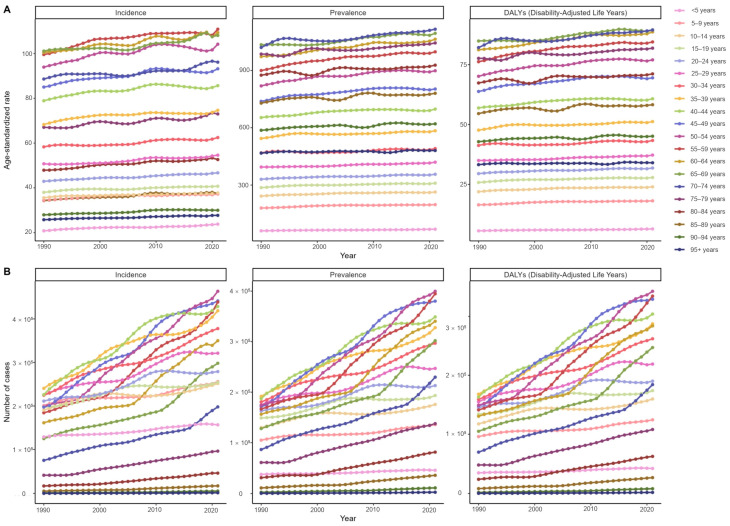
Global trends in age-standardized rates (ASRs) (**A**) and the number (**B**) of incidence, prevalence, and disability-adjusted-life-years (DALYs) of psoriasis by age from 1990 to 2021.

Analyses of age-specific psoriasis burden revealed distinct patterns across temporal and demographic dimensions. From 1990–2021, the all-age incidence and prevalence demonstrated sustained upward trajectories, mirrored by corresponding age-standardized rates (Figure 1). The EAPC analysis demonstrated a 2.36-fold variation in ASIR progression between age cohorts, with the 80–84-years age cohort (EAPC: 0.33; 95% CI [confidence interval]: 0.30–0.36) exhibiting the most notable growth and the 10–14-years one (EAPC: 0.14; 95% CI: 0.13–0.16) the least. In addition, the oldest cohort (≥95 years) displayed the most attenuated progression in prevalence (EAPC: 0.05; 95% CI: 0.03–0.08) and DALYs (EAPC: 0.04; 95% CI: 0.02–0.07), while children under five years exhibited the most accelerated rates, with both measures demonstrating EAPCs of 0.34 (95% CI: 0.31–0.36), as indicated in Supplementary Table S1.

Gender-specific analyses revealed persistent psoriasis burden disparities between 1990–2021. Males consistently demonstrated higher ASRs, with the 2021 incidence, prevalence, and DALYs being 1.02-, 1.03-, and 1.04-fold greater than females, respectively. The accelerated male progression was evidenced by higher EAPCs, 0.26 (95% CI: 0.23–0.30) for incidence, 0.27 (95% CI: 0.25–0.29) for prevalence, and 0.28 (95% CI: 0.26–0.30) for DALYs, surpassing female equivalents (0.21, 0.18, and 0.18, respectively) (Table 1).

### 3.2. Burden of Psoriasis at Regional Level

The psoriasis burden demonstrated a marked geographical heterogeneity across 54 GBD regions in 2021, reflecting distinct epidemiological patterns influenced by demographic and healthcare factors. Asia accounted for 52.3% of global incidence cases (2.8 million; 95% UI: 2.76–2.93), 51.8% of prevalent cases (22.3 million), and 52.1% of DALYs (1.9 million)—representing 109-fold-greater burdens than Australasia’s minimal counts (Supplementary Table S1). The ASR disparities showed 8.1-fold variations in ASPR and ASDR, with 5.0-fold differences in ASIR. Western Europe recorded peak ASRs (ASIR: 115.3, 95% UI: 111.9–118.9; ASPR: 1.16 thousand, 95% UI: 1.12 thousand–1.19 thousand; ASDR: 99.8, 95% UI: 72.2–133.9), followed by Andean Latin America and High-income North America. Conversely, Eastern Sub-Saharan Africa maintained the lowest ASRs (ASIR: 22.9, 95% UI: 22.1–23.6; ASPR: 150.2, 95% UI: 145.5–155.1; ASDR: 13.0, 95% UI: 9.5–17.3), with Eastern Africa and Southern Africa comprising the subsequent low-burden regions (Supplementary Figure S2).

The temporal analysis showed universal ASR increases between 1990–2021 except in Tropical Latin America. The incidence growth peaked in East Asia, North Africa and Middle East, and the Western Pacific Region, all exhibiting EAPCs exceeding 0.65. Prevalence escalation showed a concentrated intensity in East Asia and North Africa and Middle East, both surpassing EAPC thresholds of 0.80. Similarly, the DALY increases reached maximum velocity in East Asia, North Africa and Middle East, and the Western Pacific Region, each maintaining EAPCs above 0.80 (Table 1).

### 3.3. Burden of Psoriasis on National Level

**Figure 2:**
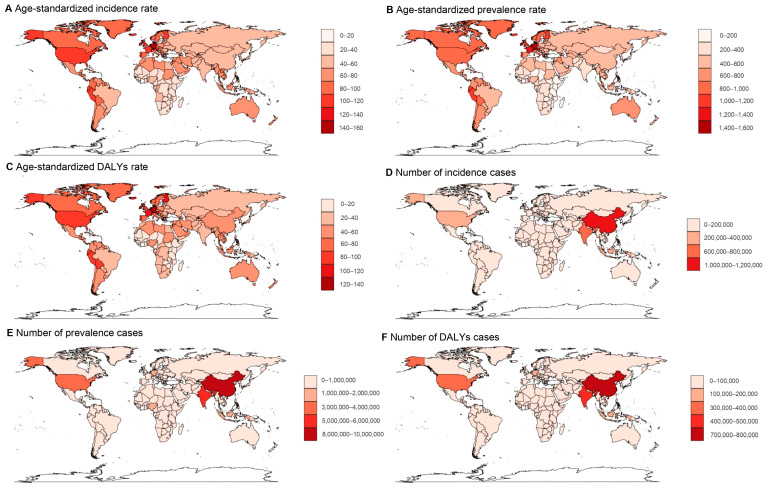
The worldwide distribution of age-standardized incidence rate (ASIR) (**A**), age-standardized prevalence rate (ASPR) (**B**) and age-standardized DALYs rate (ASDR) (**C**), along with psoriasis-related incident cases (**D**), prevalent cases (**E**), and disability-adjusted-life-years (DALYs) (**F**) for the year 2021.

**Figure 3:**
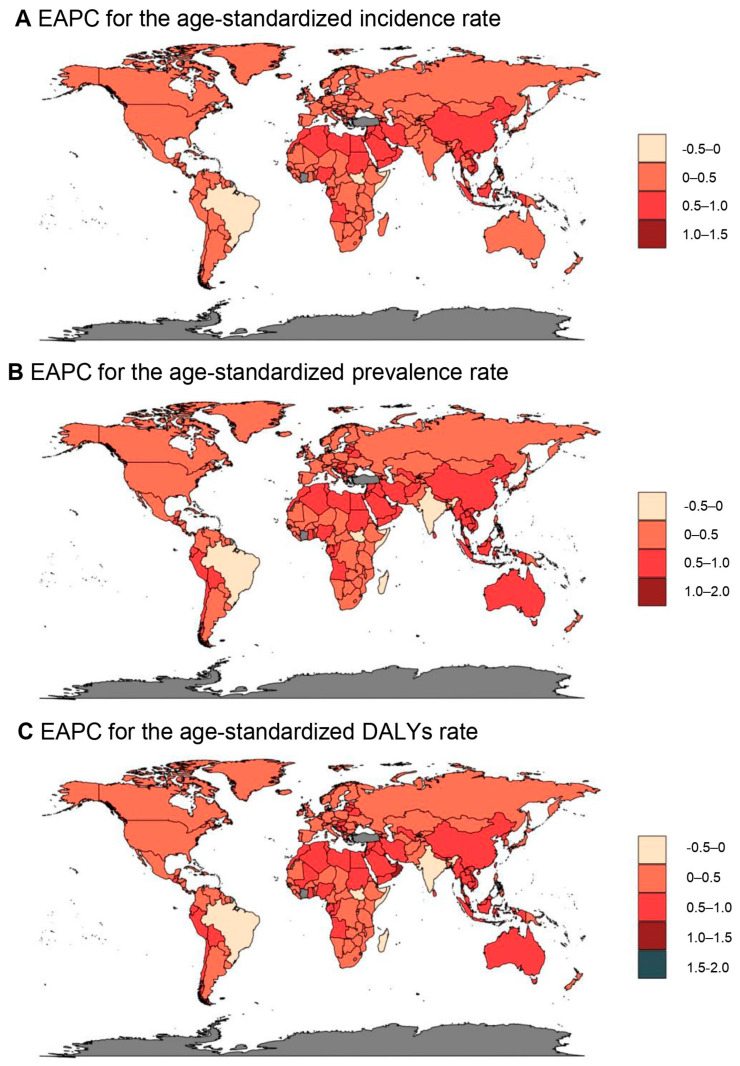
Global distribution of psoriasis estimated annual percentage changes (EAPCs) (1990–2021): (**A**) age-standardized incidence rate (ASIR), (**B**) age-standardized prevalence rate (ASPR), and (**C**) age-standardized DALYs rate (ASDR).

Figure 2 illustrates the global variation in the psoriasis disease burden on a national level. In 2021, 18 countries, including Germany, Switzerland, Monaco, San Marino, and Andorra (Figure 2C, brown, pink, and gray regions), reported an ASDR exceeding 100.0 per 100,000 person-years. Among these, Germany recorded exceptionally high figures with the highest ASIR of 143.7 (95% UI: 139.0–148.7), ASPR of 1.6 thousand (95% UI: 1.5–1.7), and ASDR of 137.5 (95% UI: 99.5–185.6), followed by Switzerland and Monaco. In contrast were countries like Somalia, Rwanda, South Sudan, Mozambique, and Burundi, among others, which reported ASDRs below 15.0 per 100,000 people in 2021. Somalia recorded the lowest rates across incidence, prevalence, and DALYs, with Rwanda close behind (Supplementary Table S1). Figure 3 portrays the global shifts in the EAPCs for psoriasis from 1990 to 2021 at the national level. During this period, Oman experienced the greatest increase in ASRs among 204 countries and territories, with an EAPC of 0.79 (95% CI: 0.75–0.83) for ASIR, 1.00 (95% CI: 0.95–1.05) for ASPR, and 1.01 (95% CI: 0.95–1.06) for ASDR, as detailed in Supplementary Table S1. The Maldives and Saudi Arabia also showed significant increases in incidence, while both Taiwan (Province of China) and Saudi Arabia had notable growth in prevalence and DALYs.

In terms of case numbers, China led with the highest counts in incidence (1.01 million, 95% UI: 0.98–1.04), prevalence (8.5 million, 95% UI: 8.2–8.7), and DALYs (728.5 thousand, 95% UI: 528.7–971.7), followed by India. Conversely, Tokelau and Niue reported near-zero figures for incidence and DALYs, with Tokelau also having the lowest prevalence figures, followed by Niue and Nauru, as shown in Supplementary Table S1.

### 3.4. Global Burden of Psoriasis by Socio-Demographic Index

The burden of psoriasis displayed a clear socioeconomic pattern across different SDI strata from 1990 to 2021. In high-SDI regions, ASRs consistently remained at their peak throughout the study period (Supplementary Figure S3A, blue fluctuating line). By 2021, these regions achieved a maximum ASIR of 92.3 (95% UI: 89.6–95.0) and an ASPR of 852.3 (95%UI: 830.1–875.3) per 100,000 individuals. Despite these high figures, the temporal progression in the disease burden within high-SDI regions was minimal (Supplementary Figure S3, Table S1). In contrast, high–middle-SDI areas exhibited the most rapid increase in ASDR, with an EAPC of 0.65 (95% CI: 0.63–0.67). Middle-SDI regions mirrored these progression patterns, albeit with a slightly reduced intensity. Notably, by 2021, middle-SDI regions (Supplementary Figure S4B, green bar) bore the largest burden of psoriasis cases. They accounted for 1.6 million (95% UI: 1.6–1.7) incidence cases, 13.3 million (95% UI: 12.8–13.7) prevalence cases, and 1.1 million (95% UI: 0.8–1.5) DALYs. Conversely, low-SDI areas (Supplementary Figure S4A, purple bar) consistently recorded the least burden across all metrics, with progressive increases in case counts observed as one moves from the low- through low–middle (red bar) to high–middle- (orange bar) SDI strata; detailed SDI values for each country during 1990–2021 are provided in Supplementary File S1.

### 3.5. The Correlation Between SDI and Psoriasis’ Incidence, Prevalence, and DALYs

**Figure 4:**
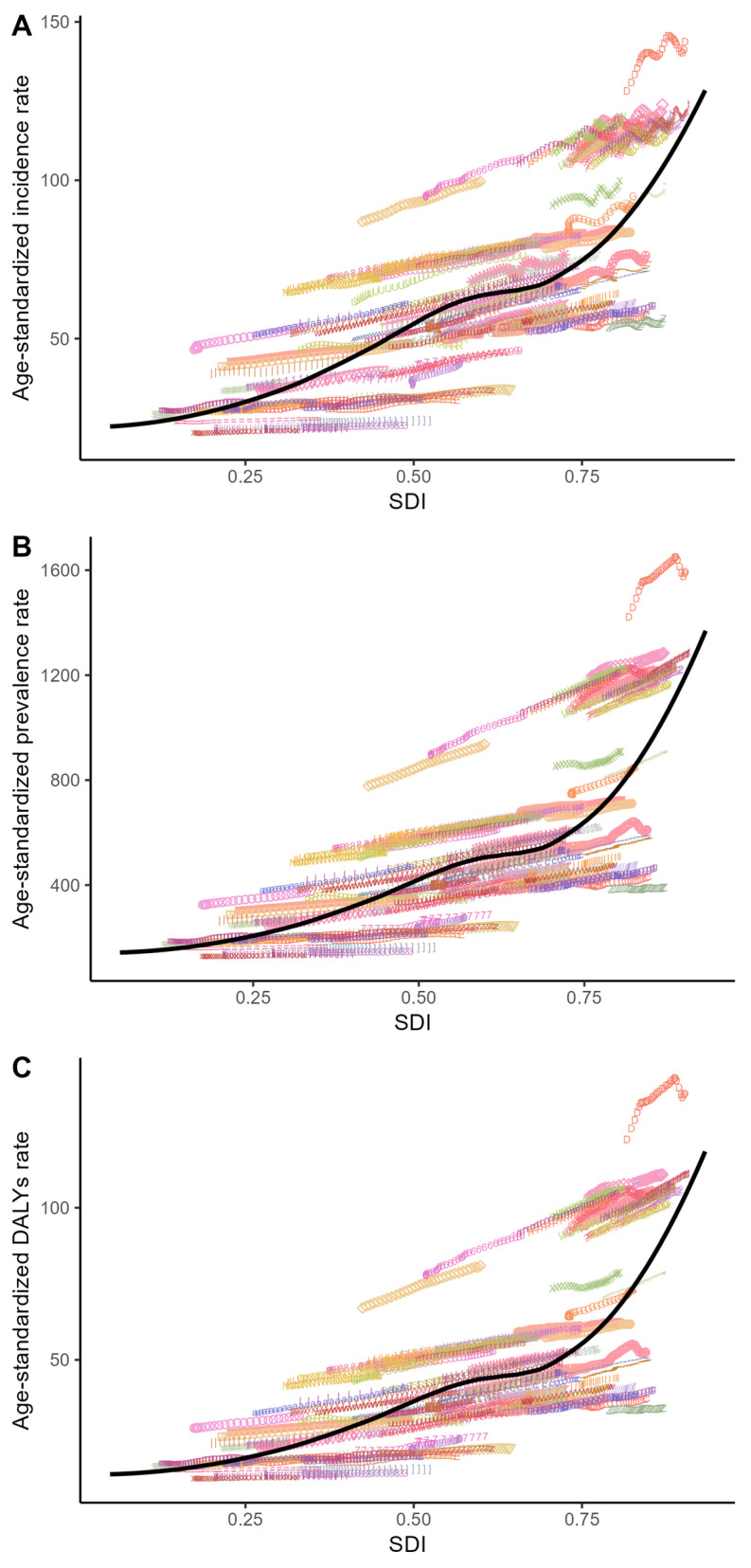
Age-standardized incidence rates (ASIRs) (**A**), age-standardized prevalence rates (ASPRs) (**B**), and age-standardized DALYs rates (ASDRs) (**C**) of psoriasis across geographic regions in 2021, stratified by the Socio-demographic Index (SDI). Each point represents one country or territory (*n* = 204); detailed listings of countries/territories within each GBD region are provided in Supplementary File S2.

**Figure 5:**
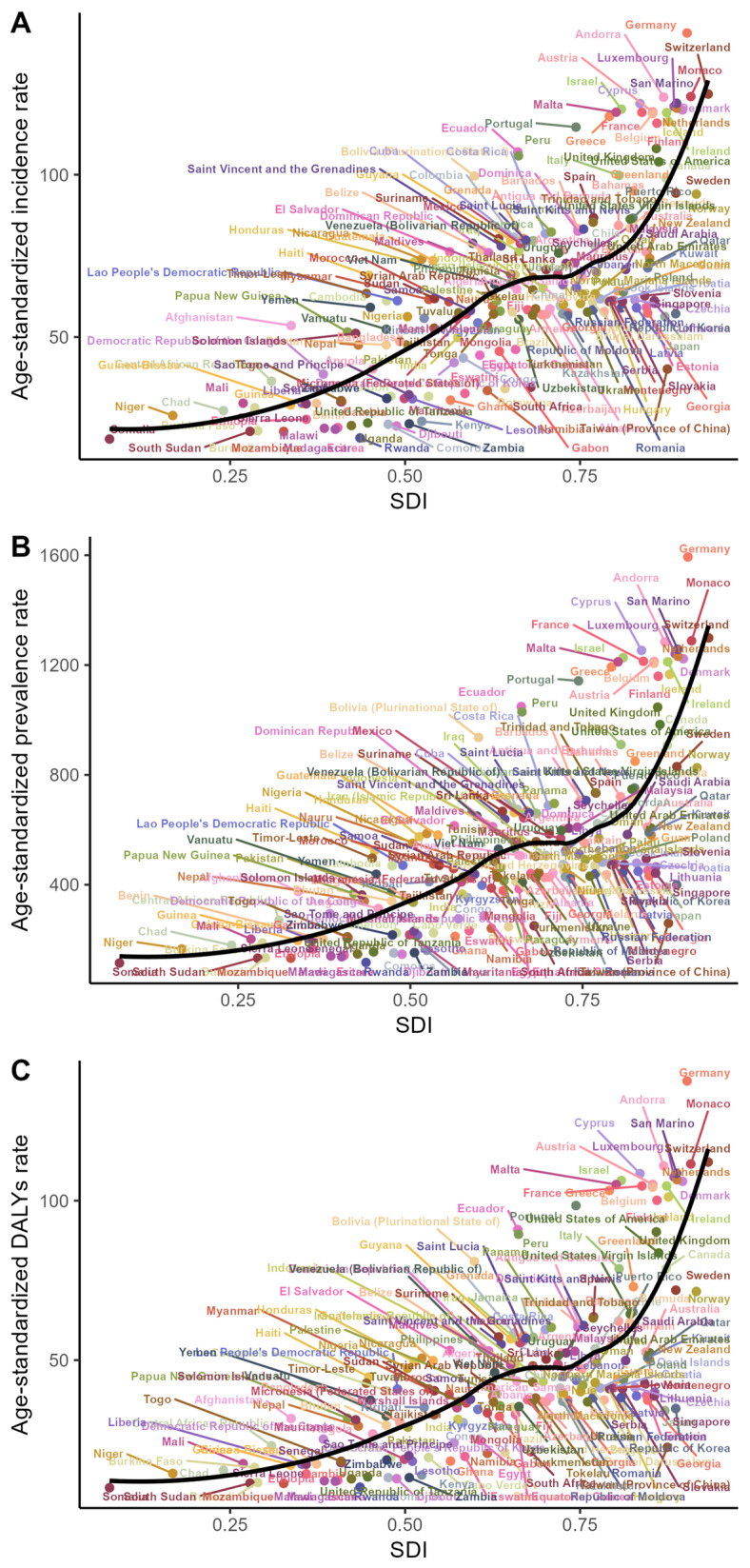
Association between age-standardized rate (ASR) and Socio-demographic Index (SDI) on national level: (**A**) trends in psoriasis age-standardized incidence rate (ASIR) by SDI; (**B**) trends in psoriasis age-standardized prevalence rate (ASPR) by SDI; and (**C**) trends in psoriasis age-standardized DALYs rate (ASDR) by SDI. Black lines across every panel denote the anticipated values for SDI and psoriasis incidence, spanning all counties.

Figure 4 presents the correlations between ASIR, ASPR, ASDR, and the SDI at the national level in 2021. A consistent positive correlation between these age-standardized rates and the SDI was observed across regions. This trend was further confirmed in Figure 5, where ASRs also increased with the rising SDI across nations. Notably, the disease burden escalated more markedly in countries with high SDI levels.

**Figure 6:**
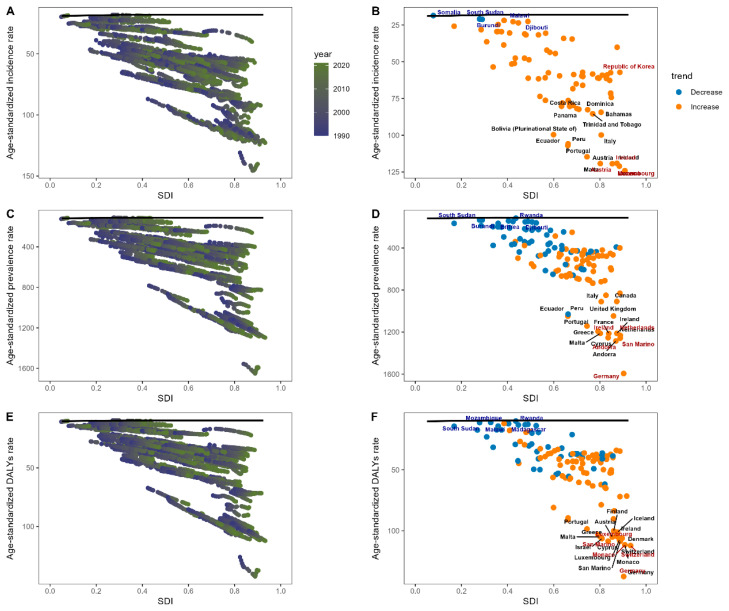
Analysis of frontier based on psoriasis-related age-standardized incidence rate (ASIR) (**A**), age-standardized prevalence rate (ASPR) (**C**), and age-standardized DALYs rate (ASDR) (**E**) in conjunction with Socio-demographic Index (SDI) spanning decades (1990–2021). The color scale transitions from dark blue in 1990 to light green in 2021, while the frontier is marked with a solid black line. In 2021, frontier analysis considering SDI (Socio-demographic Index) and psoriasis-linked ASIR (**B**), ASPR (**D**), and ASDR (**F**) is presented, with the frontier again outlined in solid black. Dots symbolize countries and territories, with the top 15 countries having the largest effective difference labeled in black. Frontier nations with low SDI (<0.5) and minimal effective difference are indicated in blue, whereas zones with high SDI (>0.85) and significant effective difference are labeled in red. Orange dots denote a reduction in psoriasis ASRs, and blue dots indicate an increase from 1990 to 2021.

To evaluate how much improvement in the ASRs of psoriasis could be achieved based on a nation’s development status, a frontier analysis using the SDI and psoriasis-related ASRs was performed across 204 countries and territories, with the results depicted in Figure 6A,C,E. The frontier line (the solid black line) marks the countries and territories with the lowest ASRs, representing the optimal performance given their SDI. The term “effective difference,” as seen in Figure 6B, Figure 6D, and Figure 6F, describes the distance from this frontier, illustrating the gap between a nation’s observed and potentially attainable levels of incidence, prevalence, and DALYs, respectively. Generally, as the SDI decreases, the effective difference tends to be smaller with less variation. For incidence, the top five countries with the highest effective difference from the frontier are Portugal, Austria, Ireland, Monaco, and Malta (Figure 6B, black text). Regarding prevalence, Malta, Ireland, Netherlands, Cyprus, and Andorra rank the highest (Figure 6D, black text). For DALYs, San Marino, Cyprus, Monaco, Switzerland, and Germany have the highest effective differences (Figure 6F, black text). These nations exhibit disproportionately higher ASRs compared to others with similar socio-demographic resources. The five countries with the lowest incidence rates, based on their development status and, thus, having the smallest effective difference, include Somalia, South Sudan, Burundi, Malawi, and Djibouti (Figure 6B, blue text). For prevalence, South Sudan, Rwanda, Burundi, Eritrea, and Djibouti (Figure 6D, blue text) are at the top. As for DALYs, Mozambique, Rwanda, South Sudan, Malawi, and Madagascar (Figure 6F, blue text) show the lowest effective difference.

## 4. Discussion

This study provides a comprehensive assessment of the global, regional, and national burden of psoriasis from 1990 to 2021, using the most recent estimates from the GBD 2021 study. Over the past three decades, we observed a notable global increase in incidence, prevalence, and DALYs attributable to psoriasis. In 2021 alone, approximately 5.1 million new individuals were affected, contributing to nearly 9% of total DALYs caused by skin diseases. The ASR of psoriasis burden showed an upward trend across most countries and territories, except for Burundi, Somalia, and South Sudan.

Importantly, our analysis revealed pronounced regional and socio-demographic disparities. ASRs increased most notably in high–middle-SDI regions, while high-SDI regions, despite consistently exhibiting the highest overall burden, demonstrated the smallest relative growth. This gradient may be attributable to the differences in healthcare access, disease awareness, and data reporting capacity. Additionally, the global prevalence of psoriasis increased by 86.4% between 1990 and 2021, accompanied by a parallel rise in total DALYs. The burden among pediatric populations increased disproportionately, indicating a potential shift in the epidemiological landscape. We also identified a clear spatial clustering of the disease burden across the 21 GBD-defined regions, further highlighting the substantial geographic heterogeneity. The observed positive correlation between the SDI and psoriasis burden may reflect both a heightened diagnostic sensitivity and an increased exposure to lifestyle-related risk factors in more developed settings. Taken together, these findings emphasize the need for regionally adapted public health strategies that are aligned with the evolving demographic, economic, and healthcare system contexts of the affected populations.

While psoriasis remains prevalent among older adults, our analysis reveals a marked escalation in both prevalence and DALYs within the pediatric population under five years of age, indicating a shifting epidemiological pattern. While older populations bear the greatest absolute burden, pediatric-onset psoriasis represents the fastest-growing subgroup. Notably, younger patients exhibit higher suicidality rates, underscoring the need for age-stratified public health strategies that combine targeted prevention for vulnerable pediatric and adolescent groups with optimized chronic disease management for aging populations [24,25].

Sex-wise, a consistently higher rate of increase in psoriasis incidence, prevalence, and DALYs has been observed among males compared to females, suggesting potential sex-based differences in biological mechanisms, environmental exposures, or healthcare utilization patterns that warrant further investigation [26]. Conversely, females report more severe psoriatic pruritus and associated psychosocial impairment, as well as distinct patterns of psoriatic arthritis characterized by peripheral joint involvement and higher pain-fatigue burdens, highlighting the need for integrated management strategies addressing psychosocial support, occupational adaptations, and sex-stratified therapeutic protocols [27,28].

Geographic and socioeconomic disparities reflect differences in healthcare access, environmental exposures, and genetic predispositions. High-SDI regions report higher psoriasis burdens, likely due to the superior diagnostic capacity, whereas middle-SDI areas exhibit rapid burden growth possibly linked to urbanization-related risks. Low-SDI regions show a lower reported burden, possibly due to underdiagnosis and a limited healthcare infrastructure [29]. Additionally, previous studies indicate that psoriasis is most prevalence among individuals of European descent, with lower occurrence rates observed in black and Hispanic populations, a pattern linked to genetic factors [30,31,32,33]. The geographic distribution of individuals of European descent partially overlaps with those of high-SDI areas, which may explain the elevated burden in these areas [30]. To further contextualize these findings, it is helpful to compare the socio-demographic distribution of psoriasis with that of other major chronic skin diseases.

Within the broader spectrum of chronic inflammatory skin diseases, the findings from GBD studies highlight both shared and divergent socio-demographic patterns. For example, atopic dermatitis (AD) exhibits the highest ASDRs in high-income or high-SDI regions, mirroring trends observed for psoriasis [34]. However, unlike psoriasis, the ASPR and ASIR for AD have globally plateaued or declined, despite the continued increases in absolute prevalence. In contrast, broader dermatitis classifications demonstrate a negative correlation between SDI and ASDRs, with lower burdens in high-SDI settings, potentially reflecting improved disease management and healthcare access [35]. Viral skin diseases similarly display an SDI-associated burden gradient, likely attributable to the enhanced diagnostic capacity and surveillance in higher-income regions [36]. Furthermore, studies on alopecia areata indicate that high-SDI regions continue to bear a substantial burden, even amid a stabilizing or declining incidence and prevalence, suggesting a complex interplay between access to care and detection bias [37]. Collectively, these comparisons suggest that, while psoriasis shares certain socio-demographic characteristics with other chronic skin conditions, its persistently increasing age-standardized burden across all SDI strata may reflect a distinct and evolving epidemiological trajectory.

The burden estimates reported in this study diverge from those presented by Damiani et al., which were based on the GBD 2019 dataset [38]. Specifically, we observed an increase in the ASIR from 57.0 in 1990 to 62.0 per 100,000 in 2021, whereas Damiani et al., using GBD 2019 data, reported a 20.0% decline in ASIR from 1990 to 2019. These discrepancies likely reflect the substantial methodological and data-related developments between GBD 2019 and GBD 2021.

Several interrelated factors may account for this divergence. First, GBD 2021 integrates over 328,000 data points—substantially more than GBD 2019—thereby providing improved demographic and geographic granularity for identifying longitudinal patterns. Second, GBD 2021 more explicitly incorporates shifting demographic structures, such as population aging and urbanization, which increasingly contribute to the psoriasis burden through behavioral and environmental risk factors. Third, it enhances the reliability of health loss estimates by refining methodologies for addressing the heterogeneity and potential biases across diverse data sources. Additionally, in our study, we applied hierarchical clustering, frontier analysis, and spatial regression to verify the internal consistency of the estimates and minimize potential analytic bias.

Several limitations should be acknowledged. First, our analysis relies exclusively on the GBD 2021 estimates, which, although robust and comprehensive, are inherently subject to modeling assumptions, data sparsity in some regions, and defined case definitions. Second, GBD does not provide subtype-specific breakdowns for psoriasis (e.g., guttate, plaque, and pustular); this limits the clinical granularity of our findings. Future studies based on clinical registries or population-level data are needed to better capture the subtype-specific disease burden and inform more tailored interventions. Third, the limited healthcare access and stigma in low-SDI regions may contribute to underdiagnosis and underreporting, leading to an underestimation of the actual disease burden. Fourth, the ecological nature of SDI-based analyses precludes causal inference at the individual level. Other potential limitations include temporal variation in the diagnostic criteria and inconsistencies between data collection methods across countries.

Despite the considerable progress in understanding the epidemiology of psoriasis, several challenges remain. One of the primary issues is the lack of consistency in the diagnostic criteria and study methodologies, which contributes to the variability in prevalence and incidence estimates [39,40]. Standardized reporting and classification criteria are necessary in order to facilitate accurate comparisons and trend analyses internationally. Addressing the current gaps demands targeted approaches such as exploring genetic–environment interactions, formulating region-specific healthcare strategies that emphasize early diagnosis and effective management [41,42], and enhancing public health campaigns tailored to high-burden regions. Moreover, digital health technologies and international data-sharing collaborations offer promising avenues for improving disease monitoring and resource allocation, particularly in resource-limited settings.

To address the rising burden of psoriasis in high-prevalence countries, the implementation of comprehensive public health strategies is essential. These strategies may include the expansion of the dermatologic care infrastructure, the integration of early screening initiatives into primary healthcare systems, and the promotion of equitable access to advanced treatments, particularly biologic agents. Additionally, large-scale patient education programs should be developed in order to enhance disease awareness and self-management. The effectiveness of these interventions will depend on their alignment with national healthcare system capacities, prevailing socioeconomic conditions, and the specific needs of target populations.

## 5. Conclusions

This study demonstrates a persistent increase in the global burden of psoriasis from 1990 to 2021, revealing significant geographic and socioeconomic disparities through comprehensive age-period-cohort analyses. These findings underscore the urgent need for regionally tailored interventions informed by localized epidemiological data to effectively mitigate the escalating global impact of psoriasis.

## Figures and Tables

**Table 1:** The number and age-standardized rate of DALYs for psoriasis globally and by GBD region in 1990 and 2021.

	1990		2021		1990–2021
Characteristics	Numbers × 10^3^ (95% UI)	ASR	Numbers × 10^3^ (95% UI)	ASR	EAPC
		No. × 10^−5^ (95%UI)		No. × 10^−5^ (95%UI)	(95% CI)
Global	1996.8 (1441.3–2670.8)	41.1 (29.8–54.9)	3689.9 (2684–4917.1)	44.4 (32.2–59.2)	0.23 (0.21–0.25)
Female	1006.7 (726.8–1342.1)	40.8 (29.5–54.4)	1812.5 (1320.4–2412.1)	43.4 (31.6–57.8)	0.18 (0.17–0.20)
Male	990.0 (714.7–1328.7)	41.4 (30–55.6)	1877.4 (1363.7–2506.6)	45.4 (33–60.6)	0.28 (0.26–0.30)
SDI					
High SDI	658.6 (477.5–882)	68.3 (49.4–91.4)	975.4 (714.2–1297.6)	73.3 (53.2–97.7)	0.18 (0.16–0.21)
High–middle SDI	418.7 (302–561.3)	39.1 (28.2–52.3)	750.4 (546.3–999.4)	47.6 (34.5–63.3)	0.65 (0.63–0.67)
Middle SDI	546.0 (393.2–729.7)	35.9 (25.9–47.9)	1144.2 (829.6–1527.3)	43.2 (31.3–57.6)	0.59 (0.57–0.61)
Low–middle SDI	286.0 (206.3–382.9)	28.9 (20.8–38.7)	599.9 (432.7–806.8)	32.5 (23.5–43.6)	0.30 (0.27–0.32)
Low SDI	85.3 (61.7–115.1)	21.2 (15.2–28.3)	216.7 (157.3–292.2)	23.1 (16.7–30.9)	0.26 (0.22–0.30)
GBD Regions					
High-income Asia Pacific	62.5 (45.5–83.7)	32.7 (23.7–43.8)	82.2 (59.7–109.3)	34.6 (24.9–46.2)	0.14 (0.13–0.16)
Central Asia	19.6 (14.1–26)	32.0 (23–42.5)	34.4 (24.9–45.8)	35.8 (25.9–47.8)	0.40 (0.38–0.43)
East Asia	351.1 (253–471.6)	31.0 (22.4–41.5)	742.9 (539.1–990.5)	40.6 (29.4–54.3)	0.89 (0.84–0.93)
South Asia	272.8 (197–366.6)	28.5 (20.6–38.2)	570.5 (410.8–765.5)	31.0 (22.4–41.7)	0.08 (0.03–0.13)
Southeast Asia	152.5 (109.9–204.8)	39.0 (28.2–52.2)	342.9 (249.7–459.2)	47.2 (34.3–63.2)	0.62 (0.60–0.63)
Australasia	9.8 (7.1–13)	45.3 (32.6–60.6)	18.8 (13.4–24.9)	52.1 (37.3–69.6)	0.56 (0.49–0.62)
Caribbean	16.6 (12–22.2)	51.2 (36.9–68.5)	26.8 (19.2–35.7)	53.7 (38.4–71.8)	0.18 (0.17–0.19)
Central Europe	48.9 (35.5–64.8)	35.9 (26.0–47.5)	58.9 (43–78.1)	41.4 (30.0–55.2)	0.49 (0.47–0.51)
Eastern Europe	83.1 (60.3–110.7)	33.4 (24.3–44.5)	95.2 (69.6–127.1)	37.9 (27.5–50.5)	0.45 (0.43–0.48)
Western Europe	396.7 (286.4–531.5)	90 (64.8–120.1)	541.5 (393.9–723.9)	99.8 (72.2–133.9)	0.26 (0.22–0.30)
Andean Latin America	24.4 (17.7–33)	74.5 (54.0–100.6)	58.2 (42.2–77.6)	88.4 (64.1–117.7)	0.59 (0.55–0.62)
Central Latin America	70.8 (50.8–94.6)	50.5 (36.4–66.8)	144.9 (104.6–193.6)	55.6 (40.1–74.3)	0.32 (0.31–0.32)
Southern Latin America	22.5 (16.3–30)	46.3 (33.6–61.7)	39.0 (28.4–52.1)	52.6 (38.3–70.5)	0.38 (0.37–0.39)
Tropical Latin America	49.2 (35.4–65.8)	35.5 (25.6–47.6)	85.3 (61.9–113.2)	35.2 (25.5–46.8)	0.04 (0.03–0.06)
North Africa and Middle East	94.0 (67.6–126.8)	34.5 (25.0–46.1)	265.4 (189.4–355)	43.6 (31.2–58.3)	0.81 (0.79–0.83)
High-income North America	241.4 (175.2–323.1)	80.4 (58.1–107.4)	362.2 (265.4–476.6)	83.1 (60.4–109.7)	0.12 (0.10–0.14)
Oceania	1.5 (1.1–2)	29.2 (21.0–38.4)	3.9 (2.8–5.2)	32.9 (23.6–43.6)	0.36 (0.35–0.38)
Central Sub-Saharan Africa	9.9 (7.1–13.3)	22.4 (16.1–29.7)	29.7 (21.6–40)	26.0 (18.8–34.9)	0.51 (0.42–0.59)
Eastern Sub-Saharan Africa	18.7 (13.5–25.2)	12.6 (9.1–16.9)	45.3 (33–60.8)	13.0 (9.5–17.3)	0.14 (0.12–0.15)
Southern Sub-Saharan Africa	8.9 (6.4–11.8)	19.8 (14.3–26.3)	16.1 (11.6–21.6)	20.8 (14.9–27.8)	0.23 (0.20–0.26)
Western Sub-Saharan Africa	41.8 (30.4–56.5)	27.1 (19.7–36.3)	125.8 (91.6–169.1)	31.6 (22.9–42.2)	0.61 (0.56–0.66)

No., number; DALYs, disability-adjusted-life-years; ASR, age-standardized rate; UI, uncertainty intervals; CI, confidence interval; SDI, Socio-demographic Index; GBD, Global Burden of Diseases; EAPC, estimated annual percentage change.

## Data Availability

The data analyzed during the current study are available in the Global Burden of Disease study 2021 (https://ghdx.healthdata.org/gbd-results-tool, accessed on 19 February 2025).

## Supplementary Materials

The following supporting information can be downloaded at: https://www.mdpi.com/article/10.3390/healthcare13192437/s1.

## Abbreviations

The following abbreviations are used in this manuscript:

GBD	Global Burden of Diseases
DALYs	Disability-Adjusted Life Years
ASR	Age-Standardized Rate
ASIR	Age-Standardized Incidence Rate
ASPR	Age-Standardized Prevalence Rate
ASDR	Age-Standardized DALY Rate
SDI	Socio-demographic Index
EAPC	Estimated Annual Percentage Change
CI	Confidence Interval
UI	Uncertainty Interval
HDI	Human Development Index
IHME	Institute for Health Metrics and Evaluation
AD	Atopic Dermatitis
GHDx	Global Health Data Exchange
