# Supplementary material for: Global Psoriasis Burden 1990–2021: Evolving Patterns and Socio-Demographic Correlates in the Global Burden of Disease 2021 Update
Source: Healthcare (Basel). 2025 Sep 26;13(19):2437. doi: 10.3390/healthcare13192437 (PMC12524270; doi:10.3390/healthcare13192437)

Supplementary Figures

Global Psoriasis Burden 1990-2021: Evolving Patterns and Socio-Demographic Correlates in the Global Burden of Disease 2021 Update

Supplementary figure legends

Supplementary Figure S1. Schematic overview of the methodological framework employed in this study.

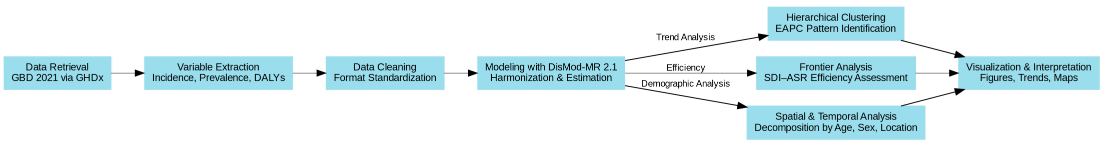

Supplementary Figure S2. Global burden of disease (GBD) region-specific analysis of psoriasis burden in 2021. age-standardized rates (ASRs) (A) and the case numbers (B) for incidence, prevalence, and disability-adjusted-life-years (DALYs) across different GBD regions.

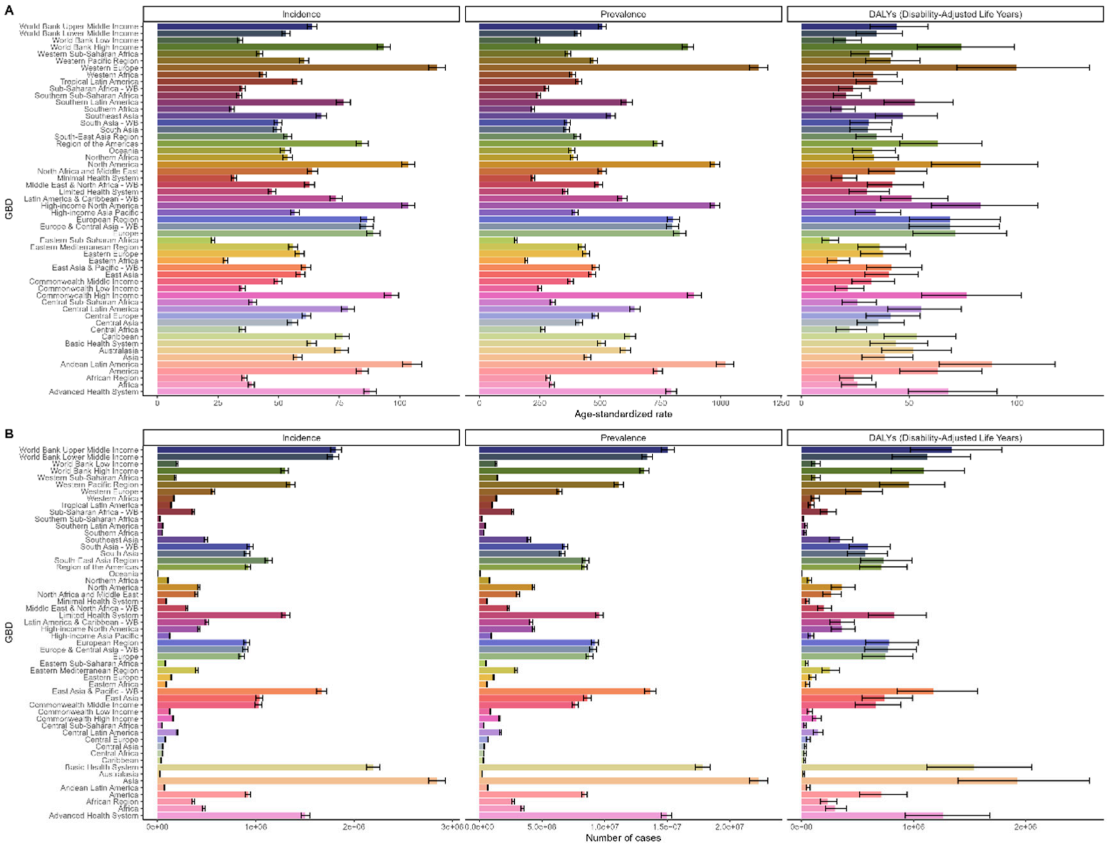

Supplementary Figure S3. Global trends in age-standardized rates (ASRs) and the number of incidence, prevalence, and disability-adjusted-life-years (DALYs) of psoriasis by socio-demographic index (SDI) from 1990 to 2021.

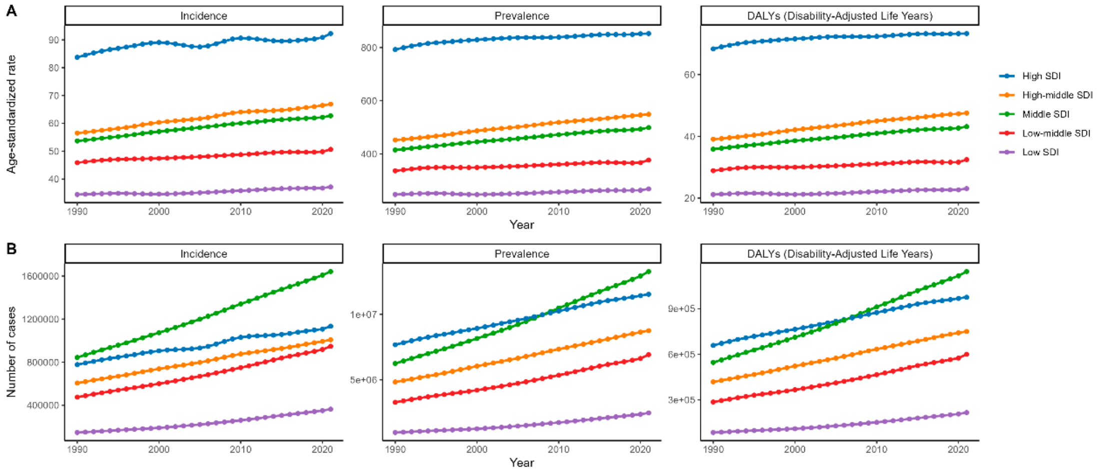

Supplementary Figure S4. Socio-demographic index (SDI)-specific incidence, prevalence, and DALYs of psoriasis in 2021. Age-standardized rates (ASRs) (A) and number of cases (B) of incidence, prevalence, and disability-adjusted-life-years (DALYs) across different SDI levels.

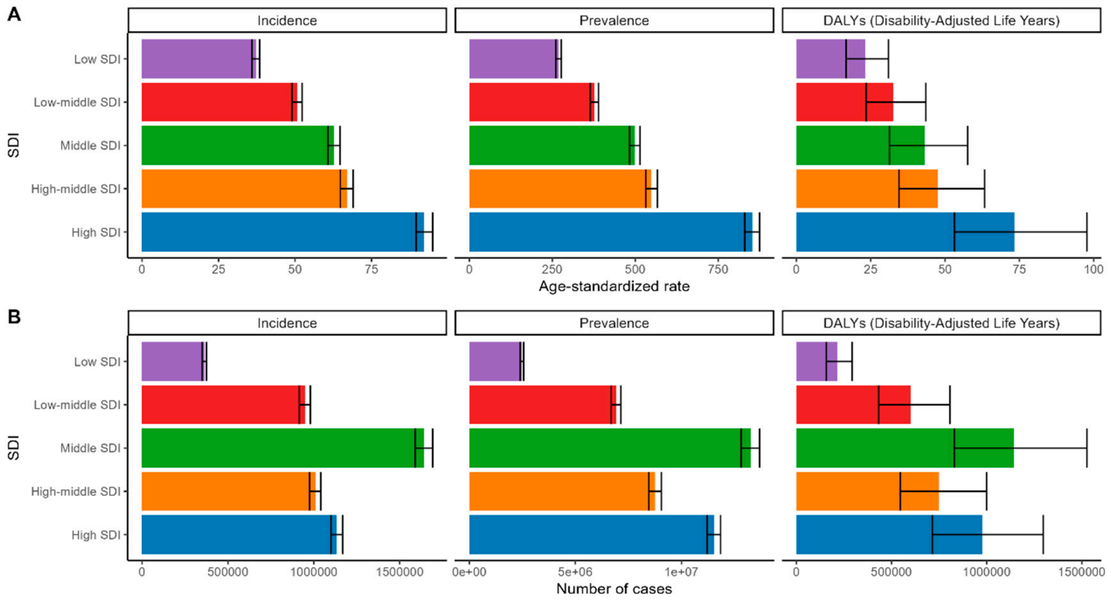

Supplement: Supplementary file 1 [file healthcare-13-02437-s001.zip › supplementary figures.pdf]
