# Supplementary material for: Global Psoriasis Burden 1990–2021: Evolving Patterns and Socio-Demographic Correlates in the Global Burden of Disease 2021 Update
Source: Healthcare (Basel). 2025 Sep 26;13(19):2437. doi: 10.3390/healthcare13192437 (PMC12524270; doi:10.3390/healthcare13192437)
Supplement: Supplementary file 1 [file healthcare-13-02437-s001.zip › supplementary file 2 21 GBD region_Country.pdf]

1. Andean Latin America
Bolivia (Plurinational State of), Ecuador, Peru

2. Australasia
Australia, New Zealand

3. Caribbean
Antigua and Barbuda, The Bahamas, Barbados, Belize, Cuba, Dominica, Dominican Republic, Grenada, Guyana, Haiti, Jamaica, Saint Lucia, Saint Vincent and the Grenadines, Saint Kitts and Nevis, Suriname, Trinidad and Tobago

4. Central Asia
Armenia, Azerbaijan, Georgia, Kazakhstan, Kyrgyzstan, Mongolia, Tajikistan, Turkmenistan, Uzbekistan

5. Central Europe
Albania, Bosnia and Herzegovina, Bulgaria, Croatia, Czechia, Hungary, Montenegro, North Macedonia, Poland, Romania, Serbia, Slovakia, Slovenia

6. Central Latin America
Colombia, Costa Rica, El Salvador, Guatemala, Honduras, Mexico, Nicaragua, Panama, Venezuela (Bolivarian Republic of)

7. Central Sub-Saharan Africa
Angola, Central African Republic, Congo, Democratic Republic of the Congo, Equatorial Guinea, Gabon

8. East Asia
China, Democratic People's Republic of Korea, Taiwan (Province of China)

9. Eastern Europe
Belarus, Estonia, Latvia, Lithuania, Republic of Moldova, Russian Federation, Ukraine

10. Eastern Sub-Saharan Africa
Burundi, Comoros, Djibouti, Eritrea, Ethiopia, Kenya, Madagascar, Malawi, Mozambique, Rwanda, Somalia, South Sudan, United Republic of Tanzania, Uganda, Zambia, Zimbabwe

11. High-income Asia Pacific
Brunei Darussalam, Japan, Republic of Korea, Singapore

12. High-income North America
Canada, Greenland, United States of America

13. North Africa and Middle East
Afghanistan, Algeria, Bahrain, Egypt, Iran (Islamic Republic of), Iraq, Jordan, Kuwait, Lebanon, Libya,

Morocco, Oman, Palestine, Qatar, Saudi Arabia, Sudan, Syrian Arab Republic, Tunisia, Turkey, United Arab Emirates, Yemen

14. Oceania

Federated States of Micronesia, Fiji, Kiribati, Marshall Islands, Papua New Guinea, Samoa, Solomon Islands, Tonga, Tuvalu, Vanuatu

15. South Asia

Bangladesh, Bhutan, India, Nepal, Pakistan

16. Southeast Asia

Cambodia, Indonesia, Lao People's Democratic Republic, Malaysia, Maldives, Myanmar, Philippines, Sri Lanka, Thailand, Timor-Leste, Viet Nam

17. Southern Latin America

Argentina, Chile, Uruguay

18. Southern Sub-Saharan Africa

Botswana, Eswatini, Lesotho, Namibia, South Africa

19. Tropical Latin America

Brazil, Paraguay

20. Western Europe

Andorra, Austria, Belgium, Cyprus, Denmark, Finland, France, Germany, Greece, Iceland, Ireland, Israel, Italy, Luxembourg, Malta, Monaco, Netherlands, Norway, Portugal, San Marino, Spain, Sweden, Switzerland, United Kingdom

21. Western Sub-Saharan Africa

Benin, Burkina Faso, Cabo Verde, Cameroon, Chad, Côte d'Ivoire, The Gambia, Ghana, Guinea, Guinea-Bissau, Liberia, Mali, Mauritania, Niger, Nigeria, Sao Tome and Principe, Senegal, Sierra Leone, Togo
