# Supplementary material for: Global Psoriasis Burden 1990–2021: Evolving Patterns and Socio-Demographic Correlates in the Global Burden of Disease 2021 Update
Source: Healthcare (Basel). 2025 Sep 26;13(19):2437. doi: 10.3390/healthcare13192437 (PMC12524270; doi:10.3390/healthcare13192437)
Supplement: Supplementary file 1 [file healthcare-13-02437-s001.zip › Supplementary Table and Legend.pdf]

Supplementary Table

Global Psoriasis Burden 1990-2021: Evolving Patterns and Socio-Demographic Correlates in the Global Burden of Disease 2021 Update

Supplementary Table

Supplementary Table S1. Geographic Disparities in ASIR, ASPR, and ASDR of psoriasis in 2021 and temporary trends, 1990-2021.

	1990						2021						1990-2021		
	Incidence		Prevalence		DALYs		Incidence		Prevalence		DALYs		ASIR	ASPR	ASDR
Characteristics	Numbers (95% UI)	ASR No. ×10-5 (95%UI)	Numbers (95% UI)	ASR No. ×10-5 (95%UI)	Numbers (95% UI)	ASR No. ×10-5 (95%UI)	Numbers (95% UI)	ASR No. ×10-5 (95%UI)	Numbers (95% UI)	ASR No. ×10-5 (95%UI)	Numbers (95% UI)	ASR No. ×10-5 (95%UI)	(95% CI) EAPC_CI	(95% CI) EAPC_CI	(95% CI) EAPC_CI
Both															
Global	2852676 (2764294-2943367)	57.01 (55.27-58.76)	23056630 (22317288-23805091)	477.72 (462.1-492.7)	1996756 (1441303-2670809)	41.08 (29.75-54.94)	5099418 (4945748-5254031)	62 (60.14-63.87)	42983446 (41654457-44313231)	515.95 (500.2-531.62)	3689928 (2684040-4917113)	44.38 (32.23-59.16)	0.23 (0.21-0.26)	0.22 (0.21-0.24)	0.23 (0.21-0.25)
Female	1436064 (1390367-1482827)	57.08 (55.29-58.87)	11708724 (11334934-12082895)	476.77 (461.68-491.78)	1006735 (726797-1342077)	40.77 (29.5-54.35)	2517860 (2443552-2595491)	61.26 (59.36-63.18)	21306970 (20652688-21941354)	508.38 (492.9-523.69)	1812535 (1320380-2412066)	43.42 (31.55-57.82)	0.21 (0.19-0.23)	0.18 (0.17-0.19)	0.18 (0.17-0.2)
Male	1416612 (1372121-1461675)	56.89 (55.19-58.65)	11347907 (10979198-11718562)	478.87 (463.04-494.61)	990021 (714695-1328732)	41.42 (30.02-55.6)	2581558 (2504067-2659371)	62.77 (60.93-64.64)	21676476 (20994941-22381305)	524.61 (508.4-541.07)	1877393 (1363660-2506551)	45.44 (33-60.64)	0.26 (0.23-0.3)	0.27 (0.25-0.29)	0.28 (0.26-0.3)
Age															
<5 years	128999 (115873-141533)	20.81 (18.69-22.83)	374595 (350692-397858)	60.42 (56.57-64.18)	34596 (24312-46543)	5.58 (3.92-7.51)	156626 (141259-171210)	23.8 (21.46-26.01)	454541 (425027-481716)	69.06 (64.58-73.19)	41970 (30157-56062)	6.38 (4.58-8.52)	0.32 (0.28-0.35)	0.34 (0.31-0.36)	0.34 (0.31-0.36)
5-9 years	200945 (176228-225949)	34.44 (30.2-38.72)	1049270 (964425-1128759)	179.81 (165.27-193.44)	95900 (68360-130625)	16.43 (11.71-22.39)	255873 (224852-286709)	37.24 (32.73-41.73)	1357884 (1250931-1460430)	197.64 (182.07-212.56)	124408 (86725-166740)	18.11 (12.62-24.27)	0.21 (0.17-0.24)	0.27 (0.23-0.32)	0.28 (0.24-0.33)
10-14 years	190603 (169733-213602)	35.58 (31.69-39.87)	1297474 (1187112-1414624)	242.21 (221.61-264.08)	117397 (82823-159536)	21.92 (15.46-29.78)	251603 (224155-282433)	37.74 (33.62-42.37)	1756479 (1609511-1907513)	263.48 (241.44-286.14)	159777 (113159-216777)	23.97 (16.97-32.52)	0.14 (0.13-0.16)	0.23 (0.2-0.26)	0.24 (0.21-0.27)
15-19 years	197382 (173321-224154)	38 (33.37-43.15)	1488407 (1365338-1614758)	286.55 (262.86-310.88)	133898 (95985-181930)	25.78 (18.48-35.03)	254204 (222978-288533)	40.74 (35.73-46.24)	1933266 (1779964-2095102)	309.83 (285.26-335.76)	173923 (126007-234624)	27.87 (20.19-37.6)	0.18 (0.16-0.2)	0.2 (0.17-0.22)	0.2 (0.18-0.23)
20-24 years	211209 (186550-239864)	42.92 (37.91-48.74)	1625724 (1498800-1753006)	330.37 (304.58-356.24)	145089 (103686-194664)	29.48 (21.07-39.56)	278975 (246122-315347)	46.72 (41.22-52.81)	2129520 (1966730-2291953)	356.61 (329.35-383.81)	190034 (136016-254865)	31.82 (22.78-42.68)	0.25 (0.24-0.27)	0.21 (0.19-0.22)	0.21 (0.2-0.23)
25-29 years	224671	50.76 (44.7-	1745822	394.43	154771	34.97	321273	54.61	2469659	419.76	219017	37.23	0.26	0.2 (0.18-	0.2 (0.19-

	(197842-255038)	57.62)	(1614390-1890288)	(364.74-427.07)	(109209-210155)	(24.67-47.48)	(283639-363854)	(48.21-61.84)	(2285745-2668485)	(388.51-453.56)	(155795-294748)	(26.48-50.1)	(0.23-0.29)	0.21)	0.22)
30-34 years	224695 (197757-251541)	58.3 (51.31-65.26)	1805988 (1674073-1951652)	468.57 (434.35-506.37)	158980 (113408-214020)	41.25 (29.42-55.53)	377728 (333343-422917)	62.49 (55.15-69.96)	2969053 (2752953-3207371)	491.17 (455.42-530.6)	261940 (187768-353014)	43.33 (31.06-58.4)	0.2 (0.17-0.23)	0.12 (0.09-0.16)	0.14 (0.1-0.17)
35-39 years	240538 (211321-267266)	68.29 (59.99-75.88)	1915996 (1774026-2068026)	543.94 (503.63-587.1)	167815 (119883-226750)	47.64 (34.03-64.37)	418702 (368252-464925)	74.65 (65.66-82.89)	3276876 (3032966-3535339)	584.25 (540.76-630.34)	287250 (204383-387997)	51.22 (36.44-69.18)	0.19 (0.15-0.23)	0.15 (0.12-0.18)	0.16 (0.13-0.19)
40-44 years	226026 (196061-255389)	78.9 (68.44-89.15)	1871728 (1731005-2014564)	653.35 (604.23-703.21)	163031 (117878-216380)	56.91 (41.15-75.53)	428504 (371475-484570)	85.66 (74.26-96.87)	3489912 (3241076-3748610)	697.63 (647.89-749.35)	303897 (217538-408753)	60.75 (43.49-81.71)	0.22 (0.18-0.27)	0.19 (0.15-0.23)	0.2 (0.16-0.24)
45-49 years	197413 (174964-222795)	85.02 (75.35-95.95)	1714173 (1591213-1855088)	738.25 (685.29-798.93)	148151 (107170-198103)	63.8 (46.15-85.32)	441294 (390830-495848)	93.2 (82.54-104.72)	3801525 (3529941-4116657)	802.85 (745.49-869.4)	329026 (239033-441157)	69.49 (50.48-93.17)	0.26 (0.22-0.3)	0.26 (0.22-0.3)	0.27 (0.23-0.31)
50-54 years	199668 (174484-225558)	93.93 (82.08-106.11)	1740832 (1610620-1866175)	818.94 (757.68-877.9)	149088 (108944-197515)	70.14 (51.25-92.92)	463451 (404656-523504)	104.16 (90.95-117.66)	3996680 (3700782-4287487)	898.29 (831.78-963.65)	342529 (247780-449704)	76.99 (55.69-101.07)	0.28 (0.22-0.34)	0.28 (0.25-0.32)	0.29 (0.25-0.32)
55-59 years	184343 (161415-208519)	99.54 (87.16-112.59)	1666066 (1553562-1795383)	899.6 (838.86-969.43)	141161 (102149-186949)	76.22 (55.16-100.94)	438732 (385401-494766)	110.87 (97.39-125.03)	3946622 (3689239-4244712)	997.31 (932.27-1072.63)	334347 (240685-438396)	84.49 (60.82-110.78)	0.29 (0.24-0.33)	0.3 (0.27-0.33)	0.31 (0.27-0.34)
60-64 years	161344 (142290-183332)	100.46 (88.59-114.15)	1561794 (1447660-1684412)	972.42 (901.36-1048.76)	130409 (93190-169794)	81.2 (58.02-105.72)	349967 (308083-398552)	109.35 (96.26-124.53)	3403369 (3160963-3667225)	1063.39 (987.65-1145.84)	283767 (203235-372412)	88.66 (63.5-116.36)	0.26 (0.23-0.29)	0.29 (0.26-0.31)	0.29 (0.26-0.31)
65-69 years	124968 (110286-139559)	101.1 (89.22-112.9)	1278679 (1187075-1376104)	1034.45 (960.34-1113.27)	104911 (75329-137886)	84.87 (60.94-111.55)	298150 (263945-332566)	108.09 (95.69-120.56)	3018313 (2806825-3246270)	1094.22 (1017.55-1176.86)	247023 (176765-321001)	89.55 (64.08-116.37)	0.21 (0.17-0.25)	0.23 (0.2-0.25)	0.22 (0.2-0.25)
70-74 years	75014 (64926-85503)	88.6 (76.69-100.99)	863715 (800409-928521)	1020.2 (945.43-1096.75)	69531 (51527-90504)	82.13 (60.86-106.9)	197905 (170790-224728)	96.15 (82.97-109.18)	2296761 (2142565-2457439)	1115.8 (1040.89-1193.86)	184153 (136806-240287)	89.46 (66.46-116.74)	0.2 (0.16-0.24)	0.21 (0.17-0.24)	0.2 (0.17-0.24)
75-79 years	41274 (36001-47458)	67.05 (58.48-77.1)	606756 (566427-650280)	985.71 (920.19-1056.41)	47790 (35073-62757)	77.64 (56.98-101.95)	96287 (83969-109748)	73.01 (63.67-83.22)	1376605 (1289005-1469438)	1043.8 (977.37-1114.19)	107993 (80095-142660)	81.88 (60.73-108.17)	0.26 (0.23-0.3)	0.18 (0.16-0.21)	0.18 (0.15-0.2)
80-84 years	16943 (14426-19732)	47.89 (40.78-55.78)	309582 (289320-332350)	875.12 (817.84-939.48)	23822 (17532-31108)	67.34 (49.56-87.93)	46108 (39431-53545)	52.65 (45.02-61.14)	812412 (761234-868099)	927.59 (869.16-991.17)	62309 (45977-80292)	71.14 (52.49-91.68)	0.33 (0.3-0.36)	0.16 (0.12-0.19)	0.14 (0.11-0.18)
85-89 years	5182 (4443-	34.29 (29.4-39.43)	110130 (101891-	728.8 (674.28-	8244 (6128-	54.55 (40.55-	17160 (14677-	37.53 (32.1-	356768 (330727-	780.3 (723.35-	26640 (19876-	58.27 (43.47-	0.32 (0.28-	0.17 (0.13-	0.16 (0.12-

	5959)		117915)	780.32)	10625)	70.31)	19728)	43.15)	380461)	832.12)	34133)	74.65)	0.35)	0.21)	0.21)
90-94 years	1198 (1005-1411)	27.95 (23.45-32.93)	25144 (23381-26918)	586.77 (545.62-628.16)	1835 (1373-2327)	42.83 (32.04-54.3)	5366 (4505-6289)	30 (25.18-35.15)	110993 (103543-118390)	620.44 (578.79-661.79)	8071 (6066-10230)	45.12 (33.91-57.19)	0.28 (0.25-0.31)	0.16 (0.13-0.19)	0.15 (0.11-0.18)
95+ years	262 (201-349)	25.77 (19.7-34.29)	4756 (4348-5138)	467.16 (427.09-504.67)	338 (255-423)	33.23 (25.03-41.53)	1511 (1146-2004)	27.72 (21.02-36.76)	26209 (23917-28332)	480.87 (438.81-519.83)	1854 (1402-2359)	34.01 (25.73-43.29)	0.22 (0.21-0.24)	0.05 (0.03-0.08)	0.04 (0.02-0.07)
SDI region															
High SDI	777403 (754268-801780)	83.73 (81.18-86.34)	7678017 (7432351-7920843)	792.52 (767.72-817.34)	658615 (477508-881959)	68.33 (49.42-91.35)	1134088 (1099752-1167976)	92.25 (89.59-94.95)	11527143 (11220648-11846449)	852.3 (830.06-875.33)	975398 (714215-1297563)	73.29 (53.16-97.71)	0.2 (0.16-0.25)	0.19 (0.17-0.21)	0.18 (0.16-0.21)
High-middle SDI	606239 (587341-625371)	56.49 (54.78-58.26)	4838956 (4675112-4998755)	452.44 (437.47-467.12)	418699 (302034-561333)	39.07 (28.21-52.31)	1008070 (975952-1040170)	66.91 (64.84-68.97)	8754846 (8467374-9050801)	549.19 (531.74-567.13)	750441 (546290-999446)	47.55 (34.5-63.29)	0.54 (0.52-0.56)	0.64 (0.62-0.65)	0.65 (0.63-0.67)
Middle SDI	843819 (816666-871347)	53.66 (52.06-55.3)	6247153 (6040105-6457367)	414.95 (401.81-427.95)	546044 (393203-729745)	35.86 (25.94-47.89)	1641222 (1590306-1691699)	62.71 (60.78-64.7)	13260806 (12813804-13691417)	499.29 (483.19-514.87)	1144223 (829568-1527294)	43.16 (31.26-57.64)	0.5 (0.48-0.52)	0.59 (0.57-0.6)	0.59 (0.57-0.61)
Low-middle SDI	475261 (459036-489690)	45.87 (44.41-47.29)	3287357 (3184246-3395065)	336.66 (325.95-346.83)	286020 (206328-382897)	28.88 (20.83-38.65)	947741 (916034-979473)	50.65 (49-52.3)	6923169 (6696095-7147195)	377.2 (364.95-389.12)	599948 (432684-806757)	32.46 (23.48-43.55)	0.27 (0.26-0.28)	0.28 (0.25-0.3)	0.3 (0.27-0.32)
Low SDI	146976 (141645-151520)	34.43 (33.31-35.58)	981297 (949018-1013691)	247.68 (239.89-255.61)	85317 (61709-115148)	21.16 (15.23-28.31)	363919 (350683-375677)	37.17 (35.94-38.38)	2480276 (2402705-2562039)	268.9 (260.45-277.38)	216728 (157310-292188)	23.09 (16.72-30.94)	0.24 (0.22-0.27)	0.23 (0.19-0.27)	0.26 (0.22-0.3)
21 Regions															
Advanced Health System	1083276 (1051640-1117333)	77.76 (75.41-80.2)	10272766 (9941074-10608870)	710.53 (687.7-733.28)	880814 (637526-1178663)	61.22 (44.22-81.8)	1505679 (1459587-1550878)	87.72 (85.13-90.34)	14943224 (14531249-15368575)	794.74 (772.92-816.76)	1265457 (926984-1681624)	68.34 (49.56-90.88)	0.31 (0.27-0.34)	0.34 (0.32-0.36)	0.34 (0.32-0.36)
Africa	184290 (177382-190056)	34.58 (33.47-35.68)	1279221 (1238288-1321529)	259.75 (251.23-267.91)	111689 (81237-151232)	22.3 (16.16-29.94)	472100 (455272-487842)	38.79 (37.5-40.03)	3428136 (3316182-3541180)	300.42 (290.65-310.13)	299383 (216419-402358)	25.82 (18.64-34.54)	0.43 (0.41-0.45)	0.53 (0.51-0.56)	0.55 (0.52-0.57)
African Region	139659 (134429-143968)	32.35 (31.3-33.4)	988229 (954910-1021728)	249.29 (241.07-257.23)	86239 (62367-116398)	21.37 (15.39-28.64)	364598 (350813-376419)	35.93 (34.75-37.09)	2686012 (2597113-2778127)	284.56 (275.27-293.97)	235011 (170366-314325)	24.47 (17.76-32.61)	0.41 (0.38-0.44)	0.5 (0.47-0.53)	0.52 (0.48-0.55)
America	553172 (535271-570510)	80.07 (77.58-82.57)	4891517 (4739930-5047802)	719.65 (697.8-742.59)	422030 (304089-562366)	61.85 (44.72-82.41)	922222 (896107-948364)	84.48 (81.96-86.94)	8392955 (8177920-8614321)	738.9 (719.73-758.74)	712696 (518443-943098)	63.22 (45.73-83.76)	0.12 (0.09-0.15)	0.1 (0.08-0.11)	0.09 (0.08-0.1)
Andean Latin	31949	92.29	277522	858.53	24351	74.46	69315	105.05	668581	1018.75	58153	88.4	0.44	0.58	0.59

America	(30728-33079)	(88.81-95.47)	(267499-287900)	(827.6-889.01)	(17725-32998)	(54.04-100.56)	(66700-71993)	(101.15-109.11)	(644024-692168)	(980.45-1053.54)	(42211-77636)	(64.07-117.73)	(0.42-0.46)	(0.55-0.61)	(0.55-0.62)
Asia	1440286 (1394961-1487507)	49.21 (47.78-50.72)	10394999 (10056213-10736190)	369.51 (357.74-381.07)	907134 (653191-1220250)	31.92 (23.1-42.85)	2842095 (2756037-2930555)	57.88 (56.15-59.69)	22299362 (21561921-23024980)	447.53 (433.13-461.63)	1925310 (1396037-2571676)	38.71 (28.03-51.7)	0.49 (0.47-0.51)	0.57 (0.55-0.6)	0.58 (0.55-0.61)
Australasia	14070 (13562-14614)	66.61 (64.07-69.09)	114112 (110298-118366)	526.93 (509.3-546.47)	9773 (7054-13011)	45.29 (32.6-60.64)	26080 (25106-27050)	75.89 (73.2-78.66)	220556 (212830-228425)	605.35 (583.19-627.54)	18758 (13430-24929)	52.12 (37.31-69.57)	0.4 (0.34-0.47)	0.56 (0.49-0.62)	0.56 (0.49-0.62)
Basic Health System	1125645 (1089239-1163854)	53.01 (51.39-54.66)	8301675 (8031302-8579283)	406.27 (393.18-418.99)	726249 (523034-972504)	35.19 (25.43-47.04)	2193068 (2125142-2260748)	63.61 (61.69-65.61)	17842706 (17228458-18439266)	505.74 (489.36-522.04)	1539653 (1118013-2056168)	43.81 (31.76-58.56)	0.6 (0.58-0.63)	0.73 (0.7-0.75)	0.73 (0.7-0.76)
Caribbean	24182 (23237-25082)	72.85 (70.03-75.54)	191071 (184387-198137)	591.2 (571.15-614.04)	16639 (11973-22188)	51.17 (36.88-68.49)	37557 (36152-38986)	76.39 (73.51-79.24)	311960 (300625-324248)	623.14 (600.77-646.69)	26796 (19201-35719)	53.69 (38.43-71.79)	0.16 (0.15-0.17)	0.19 (0.18-0.19)	0.18 (0.17-0.19)
Central Africa	18547 (17799-19228)	32.44 (31.2-33.64)	125742 (121086-130188)	238.52 (230.19-246.58)	10895 (7809-14520)	20.3 (14.69-26.78)	51407 (49255-53322)	35.11 (33.78-36.39)	356494 (343380-369707)	261.71 (252.57-270.64)	31134 (22318-42199)	22.45 (16.15-30.23)	0.31 (0.25-0.36)	0.34 (0.27-0.4)	0.37 (0.3-0.44)
Central Asia	32184 (31013-33420)	50.72 (48.91-52.65)	223965 (215501-232141)	368.99 (355.5-381.55)	19574 (14085-26020)	32 (23.03-42.48)	54029 (52020-56017)	55.72 (53.64-57.8)	395830 (380915-410263)	413.71 (398.34-428.53)	34360 (24874-45831)	35.83 (25.93-47.76)	0.35 (0.33-0.37)	0.4 (0.38-0.43)	0.4 (0.38-0.43)
Central Europe	73854 (71481-76385)	55.18 (53.42-57.03)	570399 (551116-589745)	416.04 (401.96-429.98)	48924 (35515-64848)	35.86 (26.02-47.51)	81012 (78559-83476)	61.47 (59.78-63.25)	693424 (673933-713912)	479.09 (466.21-491.64)	58899 (43017-78087)	41.42 (30.03-55.15)	0.38 (0.36-0.4)	0.47 (0.45-0.49)	0.49 (0.47-0.51)
Central Latin America	106949 (102905-110561)	72.8 (70.37-75.25)	807874 (780019-835382)	585.16 (565.96-604.52)	70759 (50798-94586)	50.48 (36.44-66.77)	203443 (196311-210199)	78.63 (75.86-81.21)	1678611 (1622121-1734179)	644.12 (622.93-665.31)	144891 (104615-193605)	55.59 (40.14-74.25)	0.25 (0.24-0.26)	0.31 (0.31-0.32)	0.32 (0.31-0.32)
Central Sub-Saharan Africa	16616 (15929-17256)	35.09 (33.77-36.44)	114891 (110532-119235)	264.21 (254.53-273.75)	9933 (7108-13278)	22.43 (16.13-29.65)	47617 (45582-49513)	39.26 (37.68-40.82)	340445 (327176-353518)	302.89 (292.3-313.71)	29712 (21555-40045)	25.96 (18.79-34.86)	0.4 (0.34-0.47)	0.47 (0.38-0.55)	0.51 (0.42-0.59)
Commonwealth High Income	101720 (98753-105042)	86.56 (83.86-89.4)	988042 (957965-1019531)	800.67 (776.31-825.97)	84881 (61052-113883)	69.23 (49.79-92.89)	157864 (152790-162942)	96.59 (93.58-99.52)	1583786 (1534379-1636375)	889.02 (860.92-918.97)	134876 (98115-178223)	76.75 (55.76-102.17)	0.26 (0.21-0.31)	0.31 (0.27-0.35)	0.31 (0.27-0.35)
Commonwealth Low Income	58449 (56365-60514)	32.95 (31.85-34.16)	376675 (362602-390289)	228.66 (220.95-236.57)	33072 (23766-44209)	19.71 (14.11-26.02)	123983 (119927-128197)	35.02 (33.93-36.16)	849453 (821745-877367)	249.37 (241.32-257.71)	74175 (53379-99884)	21.5 (15.54-28.92)	0.21 (0.2-0.21)	0.29 (0.28-0.29)	0.29 (0.29-0.3)
Commonwealth Middle Income	489422 (472733-	45.64 (44.18-	3483507 (3373685-	344.13 (332.96-	302294 (218147-	29.45 (21.27-39.6)	1027934 (994269-	49.81 (48.22-	7662234 (7416814-	377.6 (365.67-	662936 (478739-	32.44 (23.52-	0.19 (0.16-	0.13 (0.09-	0.15 (0.11-0.2)

	504665)	47.05)	3600212)	354.81)	407363)		1061353)	51.4)	7915228)	389.46)	888051)	43.34)	0.21)	0.18)	
East Asia	543655 (526680-562339)	47.02 (45.51-48.45)	4006556 (3871169-4140823)	356.82 (345.24-368.14)	351134 (252981-471594)	31 (22.4-41.5)	1037234 (1003142-1069797)	59.07 (57.24-60.92)	8619615 (8324979-8915086)	466.88 (451.82-481.49)	742940 (539071-990457)	40.61 (29.37-54.28)	0.75 (0.72-0.78)	0.89 (0.84-0.93)	0.89 (0.84-0.93)
East Asia & Pacific - WB	885235 (857779-914708)	50.11 (48.57-51.63)	6521151 (6311107-6736791)	379.16 (367.01-391.2)	570610 (410762-765073)	32.91 (23.77-44.06)	1669889 (1617695-1722336)	61.36 (59.46-63.29)	13644129 (13179113-14104691)	481.53 (466.2-496.67)	1177662 (854632-1571252)	41.88 (30.3-55.97)	0.65 (0.63-0.67)	0.78 (0.76-0.81)	0.79 (0.77-0.82)
Eastern Africa	36915 (35522-38134)	26.09 (25.24-26.95)	232750 (224618-240729)	177.59 (171.61-183.57)	20484 (14821-27669)	15.28 (11.09-20.67)	92191 (88753-95375)	28.05 (27.12-28.95)	598203 (578591-618779)	194.1 (187.59-200.37)	52578 (37617-70844)	16.72 (12.05-22.35)	0.24 (0.23-0.26)	0.29 (0.28-0.31)	0.31 (0.3-0.33)
Eastern Europe	128895 (124591-133153)	52.86 (51.21-54.65)	973375 (940379-1006453)	388.53 (375.4-400.98)	83051 (60340-110730)	33.35 (24.26-44.51)	139138 (134769-143725)	58.61 (56.8-60.57)	1126083 (1087577-1166373)	442 (427.09-456.39)	95183 (69625-127100)	37.93 (27.54-50.52)	0.36 (0.34-0.38)	0.44 (0.42-0.46)	0.45 (0.43-0.48)
Eastern Mediterranean Region	149416 (144194-154105)	46.73 (45.25-48.23)	1004739 (973580-1037259)	337.31 (326.34-348.1)	87750 (63261-118743)	29.04 (21.03-38.85)	401998 (387470-416144)	56.07 (54.11-57.87)	2929992 (2825043-3032132)	423.9 (409.55-438.09)	253831 (181965-339791)	36.34 (26.06-48.58)	0.62 (0.6-0.64)	0.78 (0.76-0.8)	0.77 (0.76-0.79)
Eastern Sub-Saharan Africa	34537 (33240-35702)	22.17 (21.44-22.91)	211936 (204467-219389)	145.92 (141.01-150.76)	18692 (13486-25189)	12.56 (9.12-16.85)	82618 (79425-85441)	22.88 (22.13-23.61)	512564 (494622-530354)	150.22 (145.45-155.11)	45262 (33035-60752)	12.97 (9.46-17.31)	0.13 (0.11-0.14)	0.11 (0.1-0.13)	0.14 (0.12-0.15)
Europe	668816 (648466-690478)	77.99 (75.58-80.52)	6440381 (6230516-6655910)	720.67 (696.15-744.21)	551526 (397997-737054)	62.06 (44.69-82.93)	855120 (827534-882214)	89.12 (86.41-91.93)	8792196 (8525607-9079625)	829.66 (803.7-856.24)	746497 (544320-995326)	71.49 (51.81-95.35)	0.36 (0.33-0.4)	0.42 (0.39-0.45)	0.43 (0.4-0.46)
Europe & Central Asia - WB	690255 (669249-712670)	76.53 (74.16-79.03)	6585463 (6369071-6805945)	704.57 (680.49-727.38)	564221 (407171-754192)	60.66 (43.7-81.05)	894599 (866182-922721)	86.31 (83.62-89.07)	9074011 (8797495-9372567)	799.37 (774.09-825.2)	771029 (562270-1026936)	68.85 (49.96-91.82)	0.32 (0.29-0.36)	0.37 (0.34-0.4)	0.38 (0.35-0.41)
European Region	696956 (675841-719585)	76.68 (74.3-79.18)	6650869 (6432277-6873493)	706.22 (682.1-729.12)	569902 (411321-761723)	60.81 (43.82-81.23)	908311 (879561-936750)	86.62 (83.92-89.39)	9215713 (8935580-9518732)	802.88 (777.48-828.86)	783227 (571257-1043392)	69.16 (50.2-92.27)	0.33 (0.3-0.36)	0.38 (0.35-0.41)	0.39 (0.36-0.42)
High-income Asia Pacific	98706 (95549-101803)	52.69 (51.02-54.3)	720462 (696722-744388)	375.6 (363.28-387.96)	62536 (45549-83685)	32.7 (23.74-43.82)	126177 (121983-130130)	56.75 (54.96-58.55)	962513 (931720-993035)	395.57 (383.13-408)	82202 (59693-109284)	34.55 (24.86-46.22)	0.13 (0.09-0.17)	0.14 (0.12-0.15)	0.14 (0.13-0.16)
High-income North America	279449 (271343-288122)	96.49 (93.65-99.43)	2819864 (2735537-2910863)	933.21 (905.55-961.65)	241413 (175175-323056)	80.35 (58.05-107.44)	422371 (410311-434102)	103.45 (100.81-106.12)	4322008 (4229371-4413972)	975.72 (956.54-996.62)	362213 (265351-476605)	83.11 (60.42-109.66)	0.11 (0.07-0.16)	0.15 (0.13-0.17)	0.12 (0.1-0.14)
Latin America & Caribbean - WB	276742 (266701-285535)	68.49 (66.16-70.79)	2097490 (2028238-2169667)	542.2 (524.46-560.11)	182861 (131264-243485)	46.75 (33.68-62.09)	503181 (486227-519844)	73.71 (71.13-76.17)	4103143 (3968118-4240466)	592.74 (573.36-612.34)	353214 (255137-470581)	51.13 (36.87-68.14)	0.22 (0.2-0.24)	0.28 (0.28-0.29)	0.29 (0.28-0.3)

Limited Health System	607493 (586655-626220)	43.38 (42.01-44.72)	4242209 (4107498-4381509)	322.45 (312.11-332.28)	368850 (266585-495198)	27.62 (19.99-37.09)	1306065 (1262862-1349139)	47.32 (45.79-48.83)	9572481 (9267786-9882105)	355.54 (344.12-366.64)	830073 (597471-1114513)	30.56 (22.07-40.9)	0.21 (0.19-0.23)	0.19 (0.15-0.22)	0.21 (0.17-0.24)
Middle East & North Africa - WB	112493 (108497-116215)	51.66 (49.98-53.35)	787772 (761728-813467)	391.71 (379.07-404.55)	68771 (49855-93004)	33.7 (24.56-45.13)	298583 (288202-309494)	62.78 (60.61-64.88)	2301765 (2215754-2384260)	493.56 (476.99-511.09)	198669 (143344-266692)	42.31 (30.54-56.75)	0.66 (0.64-0.68)	0.78 (0.76-0.8)	0.78 (0.75-0.8)
Minimal Health System	33285 (32051-34390)	29.33 (28.29-30.36)	216130 (208861-223233)	205.08 (198.35-211.7)	18782 (13389-25071)	17.51 (12.52-23.28)	90227 (86635-93311)	31.67 (30.57-32.73)	587830 (567258-609232)	222.41 (215.1-229.7)	51555 (37316-69631)	19.1 (13.83-25.6)	0.3 (0.25-0.34)	0.31 (0.25-0.36)	0.33 (0.28-0.39)
North Africa and Middle East	152645 (147189-157650)	52.69 (50.95-54.39)	1078727 (1042464-1114052)	400.59 (387.12-414.08)	94033 (67583-126814)	34.46 (24.98-46.14)	395337 (381885-409180)	63.97 (61.85-66.09)	3076685 (2962329-3188107)	508.76 (490.9-526.36)	265386 (189417-355031)	43.59 (31.22-58.25)	0.66 (0.64-0.68)	0.82 (0.8-0.84)	0.81 (0.79-0.83)
North America	279445 (271339-288119)	96.49 (93.65-99.43)	2819820 (2735491-2910814)	933.19 (905.53-961.63)	241409 (175173-323052)	80.35 (58.05-107.44)	422367 (410307-434097)	103.45 (100.81-106.12)	4321979 (4229341-4413943)	975.69 (956.51-996.59)	362210 (265351-476602)	83.1 (60.42-109.66)	0.11 (0.07-0.16)	0.15 (0.13-0.17)	0.12 (0.1-0.14)
Northern Africa	47303 (45611-48978)	45.4 (43.87-46.94)	314073 (304086-323854)	320.99 (310.53-331.19)	27448 (19933-37063)	27.69 (20.21-37.22)	110165 (106080-114173)	53.73 (51.83-55.65)	783119 (754705-811525)	391.07 (377.39-405.21)	67734 (48745-90998)	33.6 (24.22-45.04)	0.57 (0.55-0.59)	0.67 (0.65-0.69)	0.66 (0.64-0.68)
Oceania	2570 (2468-2667)	47.46 (45.62-49.33)	17187 (16530-17819)	340.32 (327.19-352.57)	1501 (1075-1980)	29.19 (21.01-38.36)	6516 (6252-6776)	52.76 (50.6-54.86)	44638 (42998-46293)	382.49 (368.67-396.23)	3902 (2786-5190)	32.94 (23.6-43.58)	0.33 (0.32-0.34)	0.36 (0.35-0.37)	0.36 (0.35-0.38)
Region of the Americas	553172 (535271-570510)	80.07 (77.58-82.57)	4891517 (4739930-5047802)	719.65 (697.8-742.59)	422030 (304089-562366)	61.85 (44.72-82.41)	922222 (896107-948364)	84.48 (81.96-86.94)	8392955 (8177920-8614321)	738.9 (719.73-758.74)	712696 (518443-943098)	63.22 (45.73-83.76)	0.12 (0.09-0.15)	0.1 (0.08-0.11)	0.09 (0.08-0.1)
South-East Asia Region	575089 (556046-593475)	48.53 (47-50.03)	4071901 (3942553-4206281)	362.78 (351.08-374.05)	354219 (255303-475425)	31.12 (22.45-41.71)	1128735 (1093500-1165979)	53.78 (52.1-55.49)	8490613 (8208474-8757965)	405.57 (392.5-418.33)	733692 (529197-985552)	34.93 (25.22-46.84)	0.23 (0.21-0.25)	0.2 (0.16-0.24)	0.22 (0.18-0.26)
South Asia	453226 (438164-467391)	45.36 (43.97-46.77)	3143242 (3043455-3246837)	332.61 (322.04-342.84)	272799 (197005-366605)	28.46 (20.64-38.19)	910977 (881352-941591)	49.4 (47.83-50.95)	6603080 (6392737-6815308)	360.98 (349.55-372.38)	570539 (410766-765530)	31.02 (22.4-41.66)	0.15 (0.13-0.18)	0.06 (0.01-0.11)	0.08 (0.03-0.13)
South Asia - WB	467004 (451628-481656)	45.66 (44.26-47.07)	3244924 (3142304-3352099)	335.42 (324.81-345.76)	281622 (203363-378518)	28.7 (20.81-38.53)	942012 (911286-973796)	49.73 (48.15-51.31)	6837231 (6618814-7055445)	364.4 (352.89-375.92)	590668 (424918-793424)	31.31 (22.59-42.06)	0.16 (0.13-0.18)	0.07 (0.02-0.12)	0.09 (0.04-0.14)
Southeast Asia	236373 (228543-244459)	58 (56.18-59.86)	1741466 (1683853-1799524)	451.86 (437.03-466.76)	152519 (109886-204778)	39.03 (28.23-52.22)	491724 (475357-507615)	67.65 (65.46-69.82)	3949656 (3809325-4084197)	545.05 (526.41-563.18)	342925 (249665-459152)	47.18 (34.33-63.17)	0.49 (0.49-0.5)	0.6 (0.59-0.61)	0.62 (0.6-0.63)
Southern Africa	23258	28.66	154500	202.76	13510	17.44	50734	30.72	346630	220.29	30076	18.81	0.28	0.31 (0.3-	0.3 (0.28-

	(22373-24012)	(27.65-29.61)	(149399-159718)	(196.23-209.3)	(9644-18144)	(12.47-23.18)	(48864-52440)	(29.68-31.72)	(335395-358540)	(213.42-227.35)	(21738-39947)	(13.67-25.02)	(0.26-0.29)	0.33)	0.32)
Southern Latin America	33084 (31784-34406)	67.7 (65.07-70.39)	259335 (250525-268897)	535.34 (517.02-555.29)	22485 (16294-29962)	46.34 (33.61-61.7)	55543 (53415-57531)	76.87 (73.86-79.68)	454728 (438288-473169)	609.49 (587.33-633.66)	39041 (28403-52060)	52.63 (38.26-70.54)	0.3 (0.25-0.34)	0.39 (0.38-0.4)	0.38 (0.37-0.39)
Southern Sub-Saharan Africa	14870 (14320-15350)	31.84 (30.73-32.9)	101562 (98049-105128)	229.68 (222.13-237.23)	8861 (6396-11753)	19.76 (14.34-26.3)	26556 (25627-27434)	33.78 (32.65-34.89)	188239 (181892-194701)	243.87 (236.15-252.15)	16135 (11579-21561)	20.76 (14.9-27.75)	0.26 (0.24-0.29)	0.26 (0.24-0.29)	0.23 (0.2-0.26)
Sub-Saharan Africa - WB	137415 (132265-141648)	31.69 (30.66-32.69)	967959 (935730-1000711)	243.1 (235.14-250.77)	84488 (61421-113903)	20.84 (15.11-27.94)	362874 (349016-374636)	35.16 (33.99-36.28)	2651330 (2563037-2743409)	276.75 (267.98-285.58)	232206 (167734-310904)	23.81 (17.21-31.7)	0.41 (0.37-0.44)	0.49 (0.45-0.52)	0.51 (0.47-0.55)
Tropical Latin America	81447 (78601-83875)	57.54 (55.66-59.26)	568589 (550164-586982)	415.57 (402.35-428.95)	49228 (35412-65766)	35.54 (25.62-47.57)	138659 (134235-143056)	57.77 (55.86-59.5)	1000423 (968021-1033148)	410.82 (397.86-424.01)	85291 (61917-113230)	35.17 (25.48-46.79)	0 (-0.02-0.02)	-0.06 (-0.07--0.04)	0.04 (0.03-0.06)
Western Africa	58267 (56196-60020)	38.52 (37.29-39.72)	452156 (437018-467750)	329.38 (318.68-340.34)	39351 (28598-53117)	28.22 (20.54-37.77)	167603 (161413-172832)	43.59 (42.17-44.95)	1343690 (1297798-1390539)	384.95 (372.69-397.87)	117860 (85927-158676)	33.19 (24.11-44.38)	0.52 (0.47-0.56)	0.61 (0.56-0.67)	0.64 (0.59-0.7)
Western Europe	430713 (417260-444548)	103.67 (100.44-106.95)	4633922 (4481456-4789029)	1042.22 (1007.77-1076.72)	396708 (286402-531456)	89.98 (64.81-120.14)	565866 (547560-584609)	115.3 (111.85-118.92)	6379167 (6182112-6597001)	1155.86 (1119.41-1194.46)	541495 (393879-723857)	99.78 (72.23-133.88)	0.22 (0.16-0.27)	0.26 (0.22-0.3)	0.26 (0.22-0.3)
Western Pacific Region	725843 (703393-750473)	49.21 (47.71-50.69)	5358827 (5183149-5537381)	371.92 (359.89-383.76)	468703 (338900-628812)	32.3 (23.4-43.24)	1353662 (1310301-1396778)	60.5 (58.63-62.4)	11115282 (10738750-11492164)	473.99 (458.71-489.03)	958373 (695796-1278599)	41.25 (29.83-55.13)	0.66 (0.64-0.69)	0.8 (0.77-0.83)	0.81 (0.77-0.84)
Western Sub-Saharan Africa	62700 (60472-64605)	37.53 (36.33-38.71)	480572 (464508-497021)	316.62 (306.32-327.08)	41845 (30391-56503)	27.14 (19.67-36.31)	181650 (175025-187328)	42.16 (40.81-43.49)	1434639 (1385778-1484214)	366.3 (354.8-378.46)	125844 (91563-169082)	31.58 (22.9-42.16)	0.49 (0.45-0.54)	0.58 (0.53-0.63)	0.61 (0.56-0.66)
World Bank High Income	917713 (890851-946495)	84.37 (81.81-87.03)	9010612 (8723184-9301069)	792.72 (767.92-818.13)	753924 (546518-1009979)	68.36 (49.44-91.43)	1294227 (1254489-1332520)	93.47 (90.76-96.21)	13162079 (12809693-13531856)	863 (839.89-886.47)	1093526 (801063-1455105)	74.24 (53.86-98.96)	0.23 (0.19-0.27)	0.24 (0.22-0.26)	0.23 (0.21-0.26)
World Bank Low Income	78308 (75464-80742)	30.71 (29.69-31.71)	504298 (488075-520940)	212.35 (205.55-219.09)	47295 (34373-63577)	18.24 (13.24-24.41)	199496 (192300-206087)	34.11 (32.99-35.25)	1320623 (1277073-1365735)	241.18 (233.39-248.7)	122891 (88404-165489)	20.73 (14.89-27.79)	0.39 (0.37-0.41)	0.47 (0.44-0.49)	0.48 (0.45-0.51)
World Bank Lower Middle Income	850974 (822381-877771)	47.78 (46.28-49.26)	6030800 (5837674-6226402)	359.29 (347.81-370.35)	507200 (366446-682826)	30.84 (22.29-41.45)	1784231 (1725712-1842711)	53.11 (51.43-54.79)	13407090 (12964249-13835051)	406.66 (393.4-419.45)	1123468 (810273-1507155)	35.01 (25.35-46.86)	0.29 (0.28-0.3)	0.31 (0.28-0.33)	0.33 (0.3-0.35)
World Bank Upper Middle Income	1002692 (970476-	52.79 (51.2-54.43)	7486988 (7244010-	404.45 (391.47-	676139 (486102-	35.02 (25.25-	1817071 (1760459-	63.74 (61.82-	15056334 (14547620-	508.96 (492.61-	1340238 (973462-	44.1 (31.94-	0.63 (0.6-0.65)	0.77 (0.74-	0.77 (0.75-0.8)

	1036103)		7738535)	417.24)	903697)	46.74)	1874293)	65.76)	15566508)	525.44)	1786204)	58.82)		0.79)	
Country															
Afghanistan	3946 (3800-4088)	46.54 (44.87-48.34)	26140 (25213-27105)	324.66 (312.79-336.93)	2238 (1559-3005)	27.56 (19.34-37.05)	13537 (12943-14057)	53.54 (51.55-55.42)	86155 (82837-89601)	375.57 (362.5-389.41)	7469 (5341-9989)	31.69 (22.82-42.1)	0.48 (0.45-0.51)	0.51 (0.45-0.56)	0.49 (0.44-0.55)
Albania	1591 (1540-1651)	51.83 (50.17-53.76)	11095 (10666-11489)	377.06 (362.97-390.49)	969 (700-1292)	32.62 (23.48-43.18)	1763 (1701-1832)	58.68 (56.64-60.82)	14315 (13801-14858)	442.58 (427.63-458.32)	1222 (861-1625)	38.28 (27.05-51.25)	0.47 (0.44-0.5)	0.59 (0.56-0.62)	0.61 (0.58-0.64)
Algeria	11638 (11188-12090)	54.78 (52.85-56.76)	82211 (79042-84994)	418.52 (402.83-432.57)	7182 (5068-9691)	36.04 (25.81-48.13)	28605 (27431-29713)	64.71 (62.13-67.13)	221318 (212325-230300)	506.02 (486.61-525.89)	19104 (13832-25649)	43.5 (31.53-58.32)	0.54 (0.53-0.56)	0.61 (0.6-0.63)	0.61 (0.59-0.63)
American Samoa	22 (22-23)	55.27 (52.98-57.39)	160 (154-166)	422.79 (406.07-438.76)	14 (10-19)	36.45 (25.84-48.38)	31 (30-33)	60.96 (58.54-63.23)	242 (233-251)	470.34 (452.93-487.93)	21 (15-28)	40.16 (29.36-53.93)	0.31 (0.3-0.32)	0.34 (0.33-0.35)	0.31 (0.3-0.33)
Andorra	66 (64-69)	113.7 (109.54-117.63)	725 (697-753)	1196.47 (1154.5-1240.6)	63 (46-85)	103.77 (75.38-139.99)	126 (122-131)	123.88 (119.76-128.31)	1448 (1395-1502)	1284.26 (1239.65-1330.76)	124 (89-164)	110.93 (79.87-148.23)	0.2 (0.16-0.24)	0.23 (0.23-0.24)	0.23 (0.22-0.23)
Angola	2984 (2849-3110)	33.74 (32.44-35.11)	20276 (19492-21091)	249.4 (240.61-258.64)	1772 (1269-2418)	21.41 (15.47-28.81)	11390 (10860-11919)	40.16 (38.54-41.88)	80918 (77638-84306)	313.06 (301.19-324.81)	7097 (5079-9524)	26.87 (19.49-36.03)	0.62 (0.58-0.67)	0.79 (0.74-0.84)	0.81 (0.75-0.86)
Antigua and Barbuda	44 (42-46)	76.4 (73.53-79.71)	360 (347-373)	632.89 (609.61-656.91)	31 (22-42)	54.95 (39.43-72.9)	81 (77-84)	83.02 (79.82-86.49)	705 (679-733)	703.2 (678.41-729.72)	60 (44-80)	60.63 (43.91-80.39)	0.28 (0.26-0.29)	0.36 (0.33-0.38)	0.35 (0.32-0.37)
Argentina	22298 (21413-23212)	67.85 (65.14-70.67)	175418 (169173-182185)	537.23 (517.88-558.27)	15215 (11051-20207)	46.57 (33.79-61.9)	36660 (35215-38003)	76.49 (73.41-79.32)	298118 (287043-310271)	604.67 (581.93-629.22)	25666 (18672-34177)	52.3 (37.98-69.53)	0.27 (0.22-0.31)	0.34 (0.33-0.35)	0.34 (0.32-0.35)
Armenia	1681 (1616-1754)	50.24 (48.26-52.34)	11891 (11424-12395)	363.24 (349.12-377.61)	1034 (741-1373)	31.45 (22.74-41.58)	1893 (1813-1967)	57.02 (54.83-59.19)	14941 (14349-15511)	427.58 (410.88-443.93)	1281 (910-1697)	37 (26.36-49.28)	0.47 (0.44-0.5)	0.6 (0.56-0.63)	0.6 (0.56-0.64)
Australia	11750 (11304-12223)	66.76 (64.18-69.38)	95560 (92178-99243)	529.23 (510.68-549.57)	8191 (5919-10862)	45.54 (32.79-60.83)	21832 (20969-22704)	76.23 (73.37-79.11)	185587 (178848-192466)	610.2 (587.82-632.96)	15781 (11267-20907)	52.54 (37.35-69.89)	0.43 (0.36-0.5)	0.59 (0.52-0.67)	0.59 (0.52-0.67)
Austria	9017 (8712-9335)	107.34 (103.66-110.95)	98567 (95214-102454)	1093.86 (1056.15-1134.98)	8437 (6115-10993)	94.43 (68.26-123.33)	12161 (11715-12616)	119.36 (115.23-123.38)	137940 (133129-142796)	1212.97 (1170.07-1255.08)	11767 (8443-15579)	105.11 (75.69-140)	0.26 (0.22-0.3)	0.33 (0.32-0.35)	0.34 (0.33-0.36)
Azerbaijan	3544 (3400-	51.65 (49.63-	25026 (23980-	378.5 (364.03-	2198 (1594-	33.01 (24.08-	6492 (6228-	56.92 (54.65-	48973 (47055-	426.3 (410.87-	4253 (3061-	37.03 (26.64-	0.38 (0.34-	0.45 (0.39-	0.46 (0.4-0.52)

	3700)	53.86)	25989)	392.78)	2977)	44.62)	6784)	59.24)	50914)	442.11)	5653)	49.49)	0.41)	0.51)	
Bahamas	193 (185-201)	80.16 (77.09-83.23)	1583 (1524-1643)	681.69 (656.51-707.82)	139 (100-186)	59.12 (42.61-78.96)	348 (334-362)	84.35 (81-87.45)	3042 (2929-3153)	720.86 (695.32-745.92)	262 (190-351)	62.18 (45.25-83.5)	0.2 (0.18-0.22)	0.24 (0.22-0.26)	0.23 (0.2-0.25)
Bahrain	273 (260-286)	58.19 (55.96-60.43)	2022 (1938-2103)	459.19 (441.32-476.28)	178 (128-238)	39.63 (28.82-52.63)	1142 (1095-1193)	68.48 (65.74-71.04)	9127 (8715-9529)	551.12 (529.74-571.62)	791 (567-1072)	47.29 (33.99-64.06)	0.56 (0.54-0.59)	0.64 (0.61-0.67)	0.63 (0.6-0.66)
Bangladesh	40315 (38836-41792)	42.66 (41.17-44.25)	265585 (255658-275330)	302.95 (292.54-313.67)	23275 (16554-31179)	26.13 (18.67-34.27)	80011 (77339-82887)	48.63 (46.99-50.32)	576489 (557167-596920)	352.51 (341.1-364.44)	50092 (35706-67899)	30.51 (21.69-41.33)	0.42 (0.41-0.43)	0.48 (0.47-0.49)	0.49 (0.48-0.51)
Barbados	201 (192-209)	80.19 (76.85-83.43)	1759 (1692-1824)	681.9 (655.72-706.76)	152 (109-205)	59.2 (42.57-80.01)	281 (269-292)	83.36 (80.28-86.61)	2565 (2463-2659)	707.62 (679.77-733.35)	219 (159-286)	61.4 (44.68-80.52)	0.12 (0.11-0.13)	0.1 (0.09-0.12)	0.1 (0.09-0.11)
Belarus	5760 (5540-6024)	51.44 (49.58-53.66)	43117 (41425-44836)	375.15 (360.83-389.46)	3693 (2610-4964)	32.33 (22.83-43.77)	6236 (5979-6502)	57.87 (55.58-60.24)	50455 (48379-52343)	435.99 (418.35-452.21)	4275 (3051-5702)	37.49 (26.96-50.78)	0.42 (0.4-0.45)	0.53 (0.5-0.55)	0.53 (0.51-0.56)
Belgium	11496 (11115-11882)	105.98 (102.66-109.68)	125287 (121073-129596)	1073.99 (1037.65-1110.59)	10754 (7735-14252)	93.03 (67.09-123.27)	15179 (14644-15716)	118.82 (115.03-122.93)	171655 (165865-177958)	1206.33 (1166.23-1247.91)	14594 (10654-19419)	104.26 (75.6-139.36)	0.28 (0.24-0.32)	0.37 (0.36-0.37)	0.36 (0.35-0.37)
Belize	113 (108-117)	69.71 (67.02-72.44)	824 (794-854)	548.23 (528.07-567.4)	73 (51-96)	47.67 (33.97-62.53)	320 (306-333)	76.72 (73.56-79.7)	2538 (2442-2637)	620.93 (598.37-643.65)	221 (160-297)	53.78 (38.9-72.09)	0.3 (0.28-0.32)	0.39 (0.37-0.41)	0.38 (0.35-0.4)
Benin	1108 (1067-1149)	27.61 (26.62-28.57)	6962 (6695-7241)	188.34 (181.76-194.89)	611 (448-821)	16.2 (11.89-21.62)	3403 (3267-3533)	29.55 (28.61-30.58)	21653 (20879-22458)	201.55 (194.89-208.76)	1912 (1362-2523)	17.44 (12.6-22.99)	0.34 (0.3-0.39)	0.34 (0.29-0.39)	0.37 (0.32-0.41)
Bermuda	49 (47-51)	78.44 (75.43-81.65)	418 (402-434)	658.79 (634.26-683.12)	36 (26-48)	57.33 (41.15-76)	62 (59-64)	83.54 (80.15-86.75)	578 (556-600)	710.79 (684.4-737.5)	49 (36-65)	61.74 (45.09-82.29)	0.23 (0.22-0.24)	0.28 (0.27-0.3)	0.28 (0.27-0.3)
Bhutan	226 (218-235)	41.18 (39.84-42.62)	1474 (1416-1530)	286.49 (276.44-296.7)	129 (93-173)	24.73 (17.85-33.21)	363 (350-376)	47.58 (45.97-49.3)	2585 (2487-2682)	340.52 (328.52-352.83)	226 (161-303)	29.6 (21.13-39.61)	0.49 (0.49-0.5)	0.6 (0.58-0.61)	0.61 (0.6-0.63)
Bolivia (Plurinational State of)	4998 (4788-5181)	86.96 (83.68-90.07)	41232 (39650-42826)	778.08 (750.55-807.14)	3602 (2613-4787)	67.09 (48.8-89.14)	11554 (11107-12029)	99.6 (95.84-103.62)	106476 (102463-110470)	936.45 (902.6-971.44)	9255 (6663-12310)	80.94 (58.54-107.44)	0.43 (0.42-0.45)	0.6 (0.58-0.62)	0.61 (0.58-0.63)
Bosnia and Herzegovina	2374 (2287-2460)	50.48 (48.73-52.21)	16918 (16315-17541)	360.4 (348.2-373.17)	1466 (1066-1948)	31.16 (22.64-41.46)	2274 (2194-2365)	59.22 (57.23-61.42)	18933 (18205-19690)	450.96 (435.66-466.72)	1600 (1170-2116)	38.8 (28.07-51.72)	0.6 (0.54-0.65)	0.81 (0.74-0.87)	0.81 (0.74-0.87)

Botswana	345 (330-359)	30.44 (29.25-31.5)	2277 (2187-2368)	215.94 (207.85-224.12)	200 (140-272)	18.61 (13.13-25.08)	798 (763-831)	34.1 (32.72-35.39)	5654 (5431-5891)	247.18 (237.64-256.58)	487 (354-655)	21.08 (15.32-28.19)	0.43 (0.4-0.45)	0.49 (0.46-0.52)	0.47 (0.44-0.5)
Brazil	79424 (76660-81803)	57.57 (55.7-59.3)	554871 (536838-572869)	415.9 (402.72-429.39)	48024 (34530-64094)	35.56 (25.66-47.55)	134575 (130267-138821)	57.76 (55.86-59.5)	971497 (940062-1003123)	410.72 (397.81-423.78)	82785 (60113-109845)	35.15 (25.5-46.73)	0 (-0.02-0.01)	-0.06 (-0.08--0.05)	-0.05 (-0.06--0.03)
Brunei Darussalam	123 (118-128)	53.74 (51.95-55.83)	834 (803-870)	387.42 (373.81-402.57)	74 (53-100)	33.73 (24.44-45)	281 (270-292)	59.13 (57.04-61.19)	1987 (1915-2069)	419.22 (404.89-435.22)	174 (124-234)	36.39 (26.05-49.17)	0.21 (0.16-0.25)	0.24 (0.23-0.26)	0.24 (0.22-0.26)
Bulgaria	5274 (5091-5474)	54.13 (52.26-56.07)	41323 (39833-42848)	403.91 (389.81-418.47)	3531 (2566-4749)	34.85 (25.08-46.77)	4774 (4597-4958)	60.73 (58.59-63.04)	41079 (39536-42739)	469.75 (453.09-487.46)	3473 (2460-4651)	40.56 (28.56-54.65)	0.39 (0.37-0.41)	0.48 (0.47-0.5)	0.49 (0.48-0.51)
Burkina Faso	2099 (2012-2178)	26.28 (25.27-27.24)	13004 (12507-13521)	175.08 (169.18-181.26)	1135 (806-1520)	15.01 (10.72-20.16)	5460 (5247-5659)	28.21 (27.22-29.2)	34066 (32836-35346)	187.98 (181.4-195.09)	3013 (2101-4131)	16.35 (11.57-22.22)	0.37 (0.32-0.42)	0.38 (0.33-0.43)	0.42 (0.37-0.48)
Burundi	988 (947-1024)	21.71 (20.91-22.51)	6008 (5794-6231)	141.09 (135.97-145.65)	530 (373-709)	12.19 (8.71-16.34)	2341 (2251-2431)	21.13 (20.36-21.84)	14023 (13537-14517)	134.18 (129.46-138.49)	1243 (879-1648)	11.6 (8.15-15.4)	-0.07 (-0.08--0.06)	-0.15 (-0.16--0.14)	-0.13 (-0.14--0.12)
Cabo Verde	86 (83-89)	28.32 (27.25-29.32)	565 (545-585)	195.67 (188.86-202.67)	50 (36-67)	17.05 (12.2-22.92)	178 (172-184)	31.7 (30.61-32.72)	1257 (1212-1304)	224.53 (216.77-232.34)	109 (76-144)	19.46 (13.71-25.74)	0.52 (0.47-0.57)	0.62 (0.56-0.68)	0.61 (0.55-0.68)
Cambodia	4283 (4109-4458)	51.74 (49.84-53.91)	29315 (28254-30402)	384.2 (370.36-399.15)	2554 (1828-3475)	32.95 (23.68-44.51)	10010 (9613-10476)	61.18 (58.72-63.91)	74440 (71638-77363)	469.12 (451.67-487.26)	6467 (4602-8484)	40.4 (28.83-53.15)	0.58 (0.56-0.59)	0.69 (0.68-0.71)	0.72 (0.7-0.74)
Cameroon	2595 (2492-2687)	29.24 (28.21-30.27)	16893 (16301-17495)	205.78 (198.64-212.68)	1481 (1066-1956)	17.72 (12.74-23.3)	8826 (8457-9148)	31.73 (30.62-32.82)	58628 (56456-60884)	224.8 (216.96-232.76)	5135 (3630-6954)	19.35 (13.94-25.99)	0.39 (0.33-0.44)	0.41 (0.35-0.46)	0.42 (0.36-0.49)
Canada	24971 (24128-25851)	87.75 (84.86-90.81)	239631 (231550-247992)	810.72 (783.04-837.64)	20709 (14894-27645)	70.36 (50.41-94.62)	41412 (39975-42799)	98.92 (95.56-102.06)	415096 (400829-430451)	908.77 (877.5-940.98)	35412 (25797-46949)	78.78 (57.53-105.54)	0.28 (0.23-0.33)	0.38 (0.37-0.39)	0.37 (0.36-0.38)
Central African Republic	796 (762-830)	33.5 (32.2-34.98)	5413 (5199-5626)	246.69 (237.18-256.33)	469 (330-629)	20.98 (14.91-28.03)	1779 (1703-1855)	36.41 (34.88-37.99)	12354 (11862-12868)	270.78 (260.71-282.1)	1076 (761-1461)	23.14 (16.53-31.22)	0.31 (0.29-0.33)	0.32 (0.31-0.33)	0.35 (0.33-0.37)
Chad	1301 (1251-1348)	25.93 (24.99-26.87)	8025 (7724-8303)	171.56 (165.26-177.4)	705 (500-963)	14.83 (10.59-19.88)	3948 (3798-4085)	27.33 (26.42-28.21)	23871 (22926-24802)	179.51 (173.59-185.59)	2102 (1495-2849)	15.45 (11.04-20.78)	0.31 (0.26-0.36)	0.3 (0.24-0.36)	0.3 (0.25-0.36)
Chile	8580	67.29 (64.8-	66108	529.88	5734	45.69	16070	77.96	133238	622.78	11381	53.59	0.37	0.51 (0.5-	0.5 (0.5-

	(8253-8923)	69.85)	(63616-68622)	(509.79-548.94)	(4071-7655)	(32.51-60.76)	(15439-16742)	(74.82-81.05)	(128184-138569)	(599.29-647.33)	(8275-15242)	(38.84-71.87)	(0.33-0.42)	0.52)	0.51)
China	530265 (513660-548626)	47.5 (45.99-48.94)	3921863 (3789618-4053307)	362.04 (350.27-373.61)	343657 (247483-461828)	31.45 (22.71-42.12)	1012635 (979127-1044617)	59.7 (57.85-61.59)	8453045 (8161389-8743685)	474.02 (458.6-488.88)	728553 (528686-971655)	41.23 (29.83-55.12)	0.75 (0.72-0.78)	0.89 (0.84-0.93)	0.89 (0.84-0.94)
Colombia	22052 (21170-22949)	74.33 (71.53-77.32)	170758 (164025-177543)	606.44 (584.47-629.8)	14960 (10561-19822)	52.45 (37.33-68.79)	41207 (39513-42890)	80.74 (77.43-83.84)	352204 (339235-365239)	674.27 (649.72-699.35)	30456 (21803-40530)	58.49 (41.73-77.97)	0.28 (0.26-0.3)	0.36 (0.33-0.38)	0.37 (0.35-0.4)
Comoros	85 (82-89)	22.25 (21.47-23.05)	526 (506-546)	145.98 (141.09-150.95)	47 (33-62)	12.66 (9.08-16.55)	163 (157-168)	23.13 (22.33-23.9)	1040 (1004-1076)	151.43 (146.28-156.38)	91 (64-123)	13.16 (9.2-17.68)	0.14 (0.13-0.15)	0.12 (0.11-0.14)	0.14 (0.12-0.16)
Congo	795 (759-827)	37.87 (36.4-39.3)	5733 (5504-5948)	294.9 (283.93-305.27)	502 (355-678)	25.39 (18.45-33.58)	2212 (2114-2302)	43.56 (41.78-45.25)	17055 (16346-17724)	353.4 (339.39-366.83)	1486 (1074-1989)	30.4 (22.05-40.89)	0.49 (0.45-0.52)	0.6 (0.56-0.63)	0.61 (0.57-0.65)
Cook Islands	9 (9-10)	54.17 (52.01-56.24)	68 (65-70)	412.19 (396.13-426.98)	6 (4-8)	35.59 (25.99-47.14)	12 (12-13)	61.74 (59.39-64.13)	100 (97-104)	481.9 (464.94-500.6)	9 (6-11)	41.27 (30.24-54.56)	0.41 (0.41-0.42)	0.49 (0.48-0.5)	0.47 (0.46-0.48)
Costa Rica	2101 (2011-2183)	75.75 (72.72-78.55)	16401 (15705-17016)	624.62 (600.24-647.26)	1441 (1028-1906)	54.2 (38.77-71.74)	4092 (3935-4251)	81.84 (78.69-84.86)	35331 (34046-36548)	688.73 (663.8-712.06)	3045 (2166-4094)	59.56 (42.31-80.47)	0.25 (0.23-0.26)	0.32 (0.31-0.32)	0.31 (0.3-0.32)
Croatia	2958 (2848-3064)	54.76 (52.81-56.77)	23011 (22148-23908)	412.59 (397.14-427.82)	1986 (1391-2662)	35.83 (25.08-48.48)	2965 (2857-3074)	61.32 (59.15-63.43)	25713 (24795-26657)	478.03 (461.12-495.81)	2179 (1601-2877)	41.35 (30.34-55.3)	0.42 (0.39-0.44)	0.52 (0.5-0.54)	0.52 (0.5-0.55)
Cuba	8126 (7783-8459)	75.38 (72.27-78.48)	67429 (64959-70258)	619.22 (596.46-644.19)	5859 (4197-7799)	53.74 (38.37-71.77)	10070 (9638-10516)	78.55 (75.38-81.72)	88182 (84581-91820)	644.88 (620.55-668.52)	7541 (5398-10044)	55.92 (40.32-74.44)	0.14 (0.12-0.16)	0.14 (0.11-0.16)	0.14 (0.11-0.17)
Cyprus	839 (808-867)	104.98 (101.18-108.5)	8601 (8306-8916)	1059.06 (1022.58-1095.62)	744 (529-992)	91.77 (65.03-122.33)	1845 (1780-1912)	121.91 (118.02-126.34)	20648 (19923-21434)	1253.46 (1208.9-1297.47)	1773 (1282-2372)	108.49 (77.79-145.56)	0.4 (0.34-0.45)	0.54 (0.49-0.58)	0.54 (0.5-0.58)
Czechia	6106 (5904-6321)	54.76 (52.93-56.65)	47779 (46105-49543)	411.75 (397.34-426.89)	4089 (2939-5473)	35.51 (25.74-47.57)	7434 (7168-7725)	61.41 (59.35-63.73)	63896 (61525-66204)	478.77 (462.14-495.54)	5416 (3836-7137)	41.4 (29.51-54.89)	0.38 (0.34-0.41)	0.46 (0.41-0.5)	0.46 (0.42-0.5)
Côte d'Ivoire	2907 (2792-3011)	28.31 (27.32-29.23)	18517 (17765-19253)	196.12 (189.13-203.17)	1619 (1136-2214)	16.72 (11.95-22.7)	7513 (7218-7795)	30.49 (29.38-31.49)	49021 (47152-51013)	211.4 (203.95-218.89)	4312 (3099-5804)	18.29 (13.28-24.48)	0.35 (0.31-0.39)	0.35 (0.3-0.39)	0.39 (0.35-0.44)
Democratic People's Republic	7008 (6754-	35.24 (34-36.49)	45058 (43445-	228.83 (220.93-	3976 (2862-	20.05 (14.48-27.1)	12648 (12199-	42.14 (40.7-	85035 (81743-	276.2 (266.13-	7378 (5238-	24.08 (17.17-	0.59 (0.58-	0.64 (0.62-	0.64 (0.61-

of Korea	7260)		46781)	236.92)	5437)		13089)	43.52)	88016)	285.62)	9818)	32.01)	0.61)	0.65)	0.66)
Democratic Republic of the Congo	11577 (11085-12059)	35.31 (33.93-36.73)	80109 (76837-83182)	266.39 (256.75-275.85)	6898 (4931-9210)	22.52 (16.05-29.88)	30831 (29464-32189)	38.58 (36.93-40.2)	219104 (210292-227526)	294.73 (283.35-305.23)	19094 (13569-26119)	25.24 (18.19-34.26)	0.32 (0.24-0.4)	0.34 (0.23-0.44)	0.39 (0.28-0.5)
Denmark	6025 (5816-6252)	108.42 (104.61-111.93)	66984 (64568-69367)	1111.95 (1071.37-1149.74)	5730 (4110-7582)	96.05 (68.89-127.21)	7803 (7535-8101)	119.94 (115.83-124.3)	88853 (85813-92104)	1222.87 (1178.16-1264.19)	7594 (5525-10020)	106.05 (76.52-140.97)	0.24 (0.2-0.28)	0.3 (0.29-0.31)	0.32 (0.31-0.33)
Djibouti	76 (73-79)	21.99 (21.26-22.72)	469 (451-487)	143.72 (138.44-148.5)	42 (29-56)	12.54 (8.81-16.75)	267 (256-276)	22.61 (21.78-23.41)	1675 (1614-1734)	146.97 (141.96-151.79)	148 (103-205)	12.83 (8.93-17.64)	0.11 (0.1-0.13)	0.09 (0.07-0.1)	0.09 (0.07-0.11)
Dominica	51 (49-53)	75.68 (72.75-78.71)	411 (396-426)	622.65 (598.99-645.06)	36 (26-48)	54.04 (38.81-72.59)	60 (57-62)	82.62 (79.21-85.66)	522 (502-543)	698.66 (672-726.77)	45 (32-59)	60.29 (43.34-79.8)	0.29 (0.27-0.31)	0.38 (0.36-0.41)	0.38 (0.35-0.4)
Dominican Republic	4431 (4248-4613)	69.32 (66.37-72.06)	32837 (31584-34082)	542.85 (523.15-563.88)	2889 (2063-3896)	47.19 (33.81-63.57)	8354 (8018-8698)	75.97 (72.97-79.19)	67023 (64496-69652)	611.52 (588.25-635.01)	5805 (4151-7681)	52.86 (37.83-69.84)	0.32 (0.31-0.33)	0.42 (0.41-0.44)	0.41 (0.39-0.43)
Ecuador	8601 (8271-8929)	94.64 (91.12-98.21)	75928 (73071-78928)	894.19 (861.18-931.07)	6681 (4733-9091)	77.69 (55.13-104.88)	19231 (18483-19947)	107 (102.71-110.93)	187642 (180412-194475)	1048.56 (1008.63-1086.77)	16309 (11689-21803)	90.92 (65.24-121.56)	0.42 (0.4-0.43)	0.55 (0.54-0.57)	0.55 (0.54-0.57)
Egypt	17802 (17081-18430)	37.06 (35.75-38.24)	106930 (103167-110596)	234.66 (226.68-242.64)	9394 (6692-12877)	20.34 (14.52-27.9)	44010 (42290-45716)	44.39 (42.7-46.09)	274834 (264649-284864)	286.86 (276.51-297.33)	24035 (17027-32801)	24.84 (17.69-33.71)	0.64 (0.61-0.66)	0.72 (0.69-0.75)	0.73 (0.69-0.76)
El Salvador	3241 (3105-3368)	68.82 (66.22-71.67)	23925 (23028-24852)	537.98 (519.68-556.78)	2085 (1492-2792)	46.32 (33.29-61.74)	4846 (4652-5051)	76.24 (73.25-79.4)	39017 (37589-40509)	615.07 (592.19-638.21)	3371 (2422-4498)	53.05 (38.09-70.6)	0.35 (0.33-0.37)	0.47 (0.44-0.49)	0.47 (0.45-0.5)
Equatorial Guinea	125 (120-130)	34.3 (32.93-35.6)	860 (827-895)	255.17 (245.57-264.81)	75 (52-101)	21.79 (15.31-29.2)	623 (594-650)	46.19 (44.35-48.02)	4820 (4609-5032)	385.49 (371.44-400.97)	421 (297-563)	32.96 (23.68-44.25)	1.13 (1.08-1.18)	1.55 (1.48-1.62)	1.6 (1.51-1.68)
Eritrea	593 (569-615)	21.22 (20.39-21.93)	3556 (3427-3685)	136.84 (132.02-141.52)	316 (222-420)	11.83 (8.53-15.79)	1284 (1237-1330)	22.13 (21.35-22.87)	7884 (7619-8154)	142.52 (137.91-147.07)	698 (506-947)	12.32 (8.94-16.6)	0.14 (0.12-0.16)	0.13 (0.1-0.15)	0.14 (0.12-0.17)
Estonia	877 (843-915)	51.88 (49.79-53.97)	6635 (6369-6908)	379.38 (365.06-394.34)	568 (402-754)	32.68 (23.28-43.63)	869 (832-905)	59.18 (56.72-61.58)	7279 (6986-7567)	451.25 (434.14-467.9)	617 (442-820)	38.92 (27.76-51.63)	0.45 (0.42-0.48)	0.57 (0.54-0.6)	0.58 (0.54-0.61)
Eswatini	199 (190-208)	29.6 (28.4-30.77)	1286 (1235-1340)	207.78 (199.78-215.79)	114 (80-152)	18.02 (12.91-23.91)	346 (332-360)	32.63 (31.39-33.95)	2358 (2263-2457)	232.52 (224.09-241.17)	204 (147-276)	19.8 (14.34-26.63)	0.37 (0.35-0.39)	0.41 (0.39-0.43)	0.35 (0.33-0.37)

Ethiopia	9747 (9365-10072)	23.6 (22.87-24.35)	60889 (58868-62973)	158.81 (153.56-163.92)	5353 (3833-7160)	13.62 (9.85-18.25)	22743 (21896-23552)	24.65 (23.87-25.43)	144590 (139453-149621)	165.88 (160.3-171.18)	12733 (9049-17132)	14.3 (10.28-19.17)	0.17 (0.15-0.2)	0.17 (0.14-0.19)	0.2 (0.17-0.23)
Fiji	336 (322-349)	51.02 (49.06-53.08)	2327 (2233-2419)	377.15 (362.95-391.95)	205 (145-274)	32.6 (23.27-43.34)	539 (516-563)	58.4 (55.92-60.91)	3991 (3843-4140)	440.58 (424.57-456.54)	343 (251-450)	37.63 (27.38-49.41)	0.43 (0.42-0.44)	0.49 (0.48-0.5)	0.48 (0.47-0.49)
Finland	5633 (5433-5825)	103.99 (100.49-107.37)	60384 (58208-62710)	1042.46 (1006.72-1080.67)	5173 (3704-6889)	89.99 (65-120.36)	7138 (6873-7404)	115.86 (111.85-119.94)	81398 (78524-84409)	1159.27 (1121.54-1200.07)	6894 (4964-9215)	100.12 (71.82-134.12)	0.27 (0.23-0.31)	0.35 (0.34-0.35)	0.35 (0.35-0.35)
France	64499 (62214-66664)	105.53 (101.94-109.05)	693448 (667217-717479)	1067.99 (1028.7-1104.28)	59403 (43460-79702)	92.17 (67.07-124.02)	86918 (83869-90068)	119.21 (115.11-123.48)	990073 (956670-1026080)	1213.8 (1170.49-1256.13)	83972 (60294-111387)	104.6 (74.37-139.38)	0.27 (0.21-0.34)	0.35 (0.29-0.41)	0.35 (0.29-0.4)
Gabon	339 (324-352)	38.43 (36.81-40.09)	2500 (2406-2597)	301.62 (290.44-313.21)	218 (153-294)	25.94 (18.6-34.56)	782 (751-816)	45.62 (43.92-47.43)	6193 (5951-6434)	378.24 (363.57-392.45)	537 (380-722)	32.38 (23.2-42.94)	0.58 (0.57-0.6)	0.74 (0.73-0.75)	0.75 (0.74-0.76)
Gambia	228 (218-236)	27.71 (26.73-28.69)	1439 (1383-1494)	189.62 (182.93-196.23)	126 (90-167)	16.33 (11.49-21.58)	626 (600-649)	30.02 (28.9-30.96)	4058 (3903-4214)	206.33 (199.4-213.54)	358 (242-478)	17.82 (12.34-23.57)	0.39 (0.34-0.44)	0.41 (0.36-0.46)	0.4 (0.35-0.46)
Georgia	3143 (3015-3264)	54.11 (51.95-56.15)	23893 (22960-24749)	404.78 (389.1-419.07)	2073 (1476-2741)	35.23 (25.09-46.42)	2297 (2206-2393)	57.4 (55.23-59.76)	18394 (17646-19058)	431.96 (415.01-447.51)	1572 (1139-2114)	37.44 (26.88-51.08)	0.23 (0.21-0.26)	0.24 (0.2-0.29)	0.23 (0.18-0.27)
Germany	112787 (108585-116791)	128.16 (123.42-132.3)	1361441 (1315244-1411198)	1421.45 (1373.09-1470.83)	116240 (83934-155490)	122.51 (88.77-164.09)	137847 (132933-142995)	143.65 (138.96-148.67)	1766752 (1706472-1831074)	1593.7 (1543.4-1652.08)	149395 (107820-198715)	137.45 (99.46-185.61)	0.24 (0.16-0.31)	0.28 (0.2-0.37)	0.28 (0.2-0.37)
Ghana	3592 (3412-3750)	27.9 (26.67-29.13)	22991 (21636-24265)	191.51 (181.63-201.92)	2019 (1438-2734)	16.55 (11.88-22.23)	9534 (9097-9963)	30.56 (29.33-31.97)	63166 (59673-66651)	212.04 (201.1-222.95)	5536 (3926-7458)	18.34 (13.18-24.9)	0.45 (0.4-0.51)	0.51 (0.44-0.57)	0.53 (0.46-0.59)
Greece	11903 (11483-12329)	105.06 (101.62-108.68)	127791 (122824-132597)	1060.44 (1019.99-1098.73)	10997 (7953-14845)	91.99 (66.46-124.02)	13567 (13060-14086)	117.97 (113.86-121.84)	157385 (151646-162858)	1193.15 (1153.81-1236.61)	13361 (9806-17767)	103.19 (75.11-137.98)	0.29 (0.24-0.34)	0.37 (0.35-0.4)	0.37 (0.35-0.4)
Greenland	46 (44-48)	83.39 (80.53-86.09)	398 (382-414)	745.18 (718.17-771.76)	34 (25-46)	64.12 (46.37-85.58)	59 (57-61)	95.02 (91.9-98.1)	540 (521-560)	849.59 (821.72-880.48)	46 (34-62)	73.03 (53.28-98.45)	0.34 (0.3-0.38)	0.48 (0.46-0.51)	0.49 (0.46-0.51)
Grenada	57 (55-59)	71.89 (69.03-74.72)	440 (424-456)	574.23 (552.61-596.28)	38 (28-51)	49.7 (36.17-66.51)	88 (84-91)	80.36 (77.25-83.38)	744 (717-772)	668.73 (644.38-692.14)	64 (47-85)	57.62 (42.05-77.01)	0.34 (0.32-0.37)	0.47 (0.43-0.51)	0.46 (0.42-0.5)
Guam	72 (69-75)	57.42 (55.1-	530 (509-	446.19	47 (33-63)	38.86	111 (106-	64.2 (61.7-	909 (875-	507.77	78 (56-	44.07	0.41	0.49	0.48

		59.56)	550)	(429.6-462.62)		(28.03-51.77)	115)	66.69)	943)	(488.89-526.25)	105)	(31.78-59.05)	(0.38-0.44)	(0.44-0.53)	(0.43-0.52)
Guatemala	4687 (4487-4883)	65.43 (62.66-67.94)	32440 (31186-33618)	496.96 (478.6-516.03)	2838 (2044-3920)	42.63 (30.88-58.08)	11029 (10558-11469)	73.56 (70.51-76.3)	84973 (81720-88100)	580.99 (559.38-601.47)	7358 (5270-9949)	49.84 (36-66.88)	0.39 (0.37-0.4)	0.52 (0.5-0.54)	0.52 (0.5-0.54)
Guinea	1404 (1352-1455)	27.09 (26.17-28.07)	8925 (8606-9248)	183.08 (176.54-189.71)	782 (566-1058)	15.82 (11.44-21.09)	3353 (3221-3491)	29.11 (28.09-30.15)	21272 (20460-22103)	196.98 (189.81-203.53)	1877 (1358-2541)	17.06 (12.34-22.72)	0.35 (0.31-0.4)	0.36 (0.31-0.4)	0.38 (0.33-0.42)
Guinea-Bissau	232 (223-240)	27.46 (26.55-28.38)	1462 (1412-1514)	186.86 (180.57-193.08)	129 (91-176)	16.13 (11.49-21.83)	525 (504-545)	29.47 (28.49-30.55)	3341 (3215-3474)	200.57 (193.76-207.42)	294 (210-396)	17.29 (12.38-23.11)	0.35 (0.31-0.39)	0.35 (0.31-0.4)	0.37 (0.32-0.41)
Guyana	503 (482-523)	72.54 (69.44-75.34)	3807 (3658-3946)	582.96 (561.46-602.49)	330 (241-442)	49.79 (36.5-66.54)	611 (586-636)	80.01 (76.74-83.18)	5006 (4815-5211)	663.35 (637.98-689.49)	429 (308-580)	56.64 (40.55-76.47)	0.29 (0.27-0.31)	0.39 (0.37-0.4)	0.39 (0.37-0.4)
Haiti	3654 (3503-3803)	64.52 (62.03-67.14)	25657 (24692-26663)	485.36 (467.7-503.77)	2218 (1578-2951)	41.45 (29.54-55.09)	8340 (8006-8697)	68.97 (66.18-71.76)	60795 (58336-63155)	524.69 (505.68-544.6)	5239 (3730-7066)	44.72 (31.92-60.19)	0.22 (0.21-0.23)	0.26 (0.25-0.27)	0.27 (0.26-0.28)
Honduras	2679 (2549-2787)	66.35 (63.67-68.9)	18794 (18060-19553)	507.77 (488.63-526.96)	1656 (1166-2208)	43.99 (31.22-58.89)	6953 (6652-7227)	73.07 (70-75.97)	52969 (50877-55009)	575.01 (554.16-595.63)	4611 (3291-6169)	49.54 (35.5-65.75)	0.32 (0.31-0.33)	0.42 (0.41-0.43)	0.41 (0.4-0.43)
Hungary	6217 (5998-6436)	54.16 (52.21-56.11)	48572 (46548-50285)	404.23 (388.02-418.38)	4145 (2916-5528)	34.81 (24.41-46.48)	6712 (6464-6951)	61.02 (58.78-63.13)	57719 (55383-60003)	473.95 (456.58-492.06)	4904 (3501-6447)	41.06 (29.08-54.07)	0.41 (0.37-0.44)	0.51 (0.48-0.54)	0.54 (0.5-0.57)
Iceland	268 (259-278)	104.52 (100.97-108.11)	2758 (2662-2860)	1052.38 (1016.79-1091.38)	239 (174-322)	91.21 (66.49-123.16)	444 (428-461)	116.29 (112.13-120.55)	4804 (4634-4987)	1165.95 (1125.73-1209.16)	413 (299-549)	101.16 (73.26-135.14)	0.26 (0.22-0.3)	0.32 (0.31-0.33)	0.33 (0.32-0.34)
India	365118 (352852-376465)	46.23 (44.75-47.69)	2564832 (2483644-2651113)	342.01 (331.05-352.72)	222182 (160989-299582)	29.21 (21.19-39.32)	712330 (689472-736325)	49.84 (48.24-51.44)	5216089 (5050769-5385384)	365.82 (354.33-377.32)	449734 (325103-604121)	31.41 (22.74-42.14)	0.09 (0.05-0.13)	-0.03 (-0.09-0.03)	-0.01 (-0.07-0.05)
Indonesia	95965 (92842-99054)	58.97 (57.19-60.76)	706922 (683317-729989)	460.22 (445.03-475.05)	62050 (44844-83189)	39.8 (28.74-53.08)	201082 (194859-207346)	69.17 (67.11-71.27)	1613493 (1559130-1667709)	560.59 (542.19-578.25)	140508 (100754-187694)	48.55 (34.97-64.84)	0.5 (0.49-0.51)	0.62 (0.6-0.64)	0.63 (0.61-0.65)
Iran (Islamic Republic of)	25363 (24537-26114)	53.26 (51.72-54.91)	174295 (168850-179960)	397.34 (384.62-410.61)	15183 (11022-20371)	34.09 (24.83-45.6)	59100 (57233-61107)	65.04 (62.99-67.07)	463717 (447608-479919)	505.28 (488.94-522.26)	39784 (28894-53304)	43.27 (31.37-57.81)	0.67 (0.63-0.7)	0.8 (0.76-0.85)	0.8 (0.75-0.84)
Iraq	9224 (8874-	61.6 (59.3-63.87)	68272 (65838-	503.77 (486.05-	5942 (4185-	43.1 (30.66-57.31)	29883 (28668-	76.33 (73.49-	244030 (234192-	655.71 (629.6-	20986 (15358-	55.69 (40.64-	0.72 (0.71-	0.89 (0.87-	0.89 (0.86-

	9558)		70826)	522.67)	7998)		31167)	79.27)	253650)	680.77)	27639)	73.45)	0.74)	0.92)	0.91)
Ireland	3791 (3654-3916)	104.46 (100.67-108.02)	38754 (37348-40056)	1051.36 (1012.34-1088.66)	3336 (2402-4428)	90.7 (65.17-120.54)	6442 (6216-6685)	119.12 (115.17-123.45)	70636 (68064-73214)	1211.85 (1169.74-1256.28)	6043 (4393-8065)	104.63 (75.89-140.4)	0.34 (0.3-0.38)	0.46 (0.44-0.47)	0.46 (0.44-0.48)
Israel	5241 (5062-5428)	108.63 (104.87-112.46)	53430 (51675-55474)	1116.67 (1079.14-1160.26)	4642 (3276-6239)	96.97 (68.43-130.45)	11659 (11264-12085)	120.18 (116.16-124.66)	122817 (118826-127041)	1226.89 (1187.47-1269.39)	10588 (7553-14264)	106.33 (75.87-143.44)	0.24 (0.2-0.28)	0.29 (0.28-0.31)	0.29 (0.28-0.31)
Italy	57782 (55915-59647)	92.07 (88.91-95.02)	574002 (554343-595210)	853.04 (824.14-882.84)	48947 (35601-65274)	73.47 (53.15-98.33)	69354 (66965-71699)	99.81 (96.57-102.96)	716291 (691304-743397)	910.21 (878.57-943.43)	60658 (44118-80699)	78.65 (56.89-105.3)	0.15 (0.11-0.2)	0.18 (0.14-0.21)	0.19 (0.16-0.23)
Jamaica	1608 (1541-1672)	74.02 (70.97-76.85)	12703 (12220-13166)	601.92 (579.13-622.61)	1111 (800-1508)	52.32 (37.71-70.82)	2313 (2224-2407)	79.69 (76.63-82.87)	19614 (18810-20317)	659.45 (632.37-682.53)	1697 (1210-2302)	57.16 (40.63-77.78)	0.24 (0.22-0.25)	0.3 (0.28-0.31)	0.29 (0.27-0.31)
Japan	75052 (72683-77409)	53.45 (51.75-55.11)	557780 (539465-576225)	382.76 (370.61-394.93)	48319 (35046-64456)	33.36 (24.15-44.61)	85416 (82686-88126)	56.3 (54.53-58.06)	660535 (638987-680859)	391.23 (379.03-403.42)	56240 (40726-75023)	34.19 (24.69-45.72)	0.06 (0.02-0.1)	0.03 (0.02-0.05)	0.04 (0.02-0.06)
Jordan	1711 (1641-1775)	56.36 (54.12-58.38)	11943 (11502-12389)	437.75 (421.83-454.97)	1052 (744-1408)	37.81 (27.16-49.86)	7945 (7604-8255)	66.03 (63.47-68.33)	60548 (57878-63068)	521.72 (500.48-542.1)	5259 (3841-6996)	44.83 (32.71-59.3)	0.54 (0.53-0.56)	0.61 (0.59-0.63)	0.6 (0.58-0.62)
Kazakhstan	8150 (7816-8489)	51.55 (49.39-53.6)	58022 (55650-60095)	376.99 (361.82-390.4)	5056 (3571-6685)	32.66 (23.04-43.13)	11137 (10700-11601)	57.16 (54.89-59.49)	83229 (79781-86414)	429.29 (412.22-445.43)	7198 (5251-9557)	37.13 (27.13-49.16)	0.37 (0.35-0.38)	0.44 (0.42-0.45)	0.44 (0.42-0.46)
Kenya	4337 (4175-4479)	23.24 (22.5-23.99)	27057 (26164-28014)	156.59 (151.76-161.55)	2402 (1729-3215)	13.55 (9.75-18.14)	10669 (10284-11027)	24.04 (23.25-24.79)	68606 (66368-70928)	161.48 (156.32-166.48)	6044 (4369-8130)	13.96 (10.17-18.79)	0.11 (0.1-0.12)	0.09 (0.08-0.1)	0.11 (0.09-0.13)
Kiribati	31 (30-32)	49.67 (47.86-51.54)	211 (204-219)	362.04 (349.36-375.95)	18 (13-25)	30.94 (22.19-41.02)	62 (59-64)	55.78 (53.52-57.85)	434 (417-450)	411.18 (396.61-426.53)	38 (26-51)	35.17 (24.88-47.34)	0.37 (0.36-0.38)	0.4 (0.39-0.41)	0.41 (0.4-0.42)
Kuwait	977 (937-1024)	61.03 (58.76-63.47)	7409 (7097-7749)	494.71 (476.34-513.78)	653 (467-867)	42.89 (30.56-57)	3675 (3507-3848)	71.28 (68.51-73.96)	30635 (29077-32058)	589.67 (566.54-611.56)	2658 (1884-3559)	50.74 (36.22-67.74)	0.55 (0.53-0.56)	0.63 (0.61-0.64)	0.6 (0.59-0.62)
Kyrgyzstan	2046 (1967-2126)	50.71 (48.73-52.74)	14145 (13592-14679)	368.14 (353.9-382.12)	1241 (899-1666)	32.01 (23.39-42.92)	3608 (3454-3760)	54.35 (52.11-56.59)	25467 (24465-26470)	397.92 (382.31-413.61)	2224 (1572-2961)	34.53 (24.48-46.08)	0.23 (0.22-0.25)	0.24 (0.2-0.27)	0.24 (0.21-0.28)
Lao People's Democratic Republic	1758 (1685-1833)	51.15 (49.18-53.32)	12069 (11625-12536)	377.35 (363.89-392.9)	1057 (758-1435)	32.55 (23.37-43.74)	4215 (4048-4392)	61.18 (58.86-63.79)	31123 (29953-32292)	470.84 (453.12-487.7)	2733 (1969-3688)	40.9 (29.59-55.03)	0.61 (0.59-0.62)	0.76 (0.74-0.77)	0.79 (0.77-0.81)

Latvia	1513 (1453-1573)	52.45 (50.42-54.46)	11560 (11109-12007)	385.82 (371.43-399.99)	985 (716-1318)	33.17 (24.1-44.55)	1254 (1201-1304)	59 (56.54-61.46)	10519 (10099-10926)	448.89 (432.58-465.7)	891 (636-1186)	38.77 (27.81-51.92)	0.41 (0.37-0.44)	0.5 (0.47-0.54)	0.53 (0.49-0.56)
Lebanon	1514 (1459-1567)	54.31 (52.26-56.33)	11119 (10704-11579)	413.7 (398.15-430.73)	961 (691-1286)	35.53 (25.66-47.84)	3775 (3634-3928)	65.24 (62.88-67.84)	30176 (29016-31380)	512.41 (493.73-532.01)	2571 (1864-3421)	43.68 (31.76-58.23)	0.63 (0.62-0.65)	0.75 (0.73-0.76)	0.73 (0.72-0.75)
Lesotho	379 (363-394)	28.16 (27.02-29.28)	2468 (2370-2565)	194.14 (186.35-201.5)	217 (154-290)	16.85 (12.1-22.34)	541 (517-564)	30.98 (29.73-32.22)	3658 (3518-3806)	216.27 (208.45-224.82)	314 (222-422)	18.29 (13.11-24.41)	0.38 (0.35-0.4)	0.41 (0.39-0.43)	0.34 (0.32-0.36)
Liberia	584 (561-604)	27.84 (26.89-28.81)	3736 (3608-3879)	190.85 (184.31-197.57)	323 (229-435)	16.2 (11.36-21.49)	1446 (1391-1503)	29.78 (28.8-30.85)	9382 (9016-9705)	203.85 (196.29-210.67)	816 (570-1099)	17.37 (12.36-23)	0.39 (0.33-0.46)	0.42 (0.35-0.49)	0.44 (0.37-0.52)
Libya	1925 (1847-1995)	54.94 (52.92-56.99)	13516 (13015-14015)	420.01 (404.34-435.4)	1188 (843-1587)	36.34 (25.85-48.15)	4838 (4652-5042)	66.29 (63.95-68.88)	38162 (36532-39806)	524.77 (505.29-545.46)	3288 (2368-4458)	44.96 (32.47-60.35)	0.64 (0.56-0.71)	0.76 (0.66-0.87)	0.73 (0.63-0.84)
Lithuania	2041 (1957-2126)	52.29 (50.16-54.26)	15417 (14815-16024)	383.95 (368.96-398.8)	1322 (957-1737)	33.1 (23.82-43.54)	1879 (1798-1960)	60.38 (58.08-62.82)	15986 (15334-16608)	464.79 (446.69-482.12)	1348 (986-1817)	39.97 (29.09-54.21)	0.51 (0.48-0.53)	0.65 (0.63-0.68)	0.66 (0.64-0.69)
Luxembourg	453 (438-468)	108.76 (105.24-112.33)	4989 (4816-5171)	1117.87 (1079.93-1158.05)	428 (306-567)	96.6 (69.05-128.59)	881 (851-913)	121.04 (117.22-125.24)	9825 (9461-10219)	1241.56 (1199.59-1287.37)	842 (607-1121)	107.42 (77.86-143.69)	0.26 (0.22-0.3)	0.33 (0.32-0.33)	0.34 (0.33-0.35)
Madagascar	2136 (2049-2213)	21.66 (20.86-22.38)	13028 (12567-13525)	140.62 (135.69-145.27)	1150 (819-1602)	12.16 (8.72-17.05)	5317 (5085-5498)	21.6 (20.8-22.29)	32226 (30922-33392)	138.02 (133.02-142.84)	2866 (1988-3827)	12.02 (8.23-15.93)	0.01 (0-0.03)	-0.04 (-0.06--0.03)	-0.01 (-0.03-0)
Malawi	1710 (1645-1772)	21.37 (20.6-22.14)	10349 (9975-10746)	138.13 (133.64-142.99)	914 (656-1212)	11.93 (8.64-15.89)	3595 (3438-3734)	21.89 (21.12-22.67)	21894 (21058-22715)	140.54 (135.64-145.49)	1940 (1368-2558)	12.17 (8.68-16.06)	0.13 (0.11-0.16)	0.12 (0.09-0.14)	0.15 (0.12-0.18)
Malaysia	9696 (9311-10106)	62.87 (60.44-65.34)	73808 (70957-76675)	511.04 (491.32-531.29)	6480 (4605-8560)	44.22 (31.83-58.55)	23679 (22654-24646)	72.85 (69.68-75.74)	196569 (189067-204024)	609.86 (586.85-632.09)	17082 (12312-22642)	52.78 (38.01-69.74)	0.48 (0.46-0.51)	0.59 (0.55-0.63)	0.59 (0.56-0.63)
Maldives	96 (92-100)	54.95 (52.8-57.02)	671 (645-697)	420 (404.08-436.39)	59 (43-80)	36.42 (26.16-48.34)	380 (363-398)	69.3 (66.6-71.99)	2915 (2792-3047)	556.82 (535.02-579.21)	257 (183-346)	48.56 (35.07-64.91)	0.76 (0.74-0.78)	0.92 (0.88-0.96)	0.95 (0.9-0.99)
Mali	2012 (1909-2101)	27.37 (26.11-28.57)	12723 (12006-13413)	186.07 (176.11-196.17)	1111 (773-1465)	16 (11.25-21.09)	5953 (5668-6246)	29.63 (28.32-30.89)	37544 (35471-39656)	202.33 (191.62-213.19)	3323 (2346-4488)	17.52 (12.62-23.3)	0.44 (0.38-0.5)	0.48 (0.41-0.55)	0.5 (0.43-0.58)
Malta	408 (394-	104.93	4252 (4104-	1056.7	367 (266-	91.48 (66.3-	593 (572-	119.25	6888 (6649-	1211.83	587 (420-	105.06	0.33	0.44	0.43

	422)	(101.51-108.23)	4404)	(1020.8-1094.39)	484)	120.56)	614)	(115.3-123.19)	7156)	(1171.54-1256.93)	782)	(75.52-140.02)	(0.29-0.37)	(0.42-0.45)	(0.42-0.45)
Marshall Islands	17 (16-18)	49.32 (47.34-51.21)	113 (109-118)	357.22 (343.85-371.09)	10 (7-13)	30.8 (22.44-40.47)	30 (29-31)	55.62 (53.39-57.84)	211 (203-219)	410.24 (395.05-425.66)	18 (13-24)	35.07 (25.31-46.3)	0.35 (0.34-0.37)	0.4 (0.39-0.42)	0.39 (0.37-0.4)
Mauritania	504 (484-522)	28.55 (27.53-29.46)	3280 (3164-3404)	198.43 (191.45-205.42)	289 (203-391)	17.2 (12.17-22.82)	1206 (1159-1249)	31.06 (29.95-32.16)	7988 (7690-8291)	217.58 (209.57-225.19)	706 (499-945)	18.91 (13.56-25.19)	0.4 (0.35-0.44)	0.42 (0.37-0.48)	0.44 (0.39-0.49)
Mauritius	620 (594-646)	60.43 (58.02-63)	4755 (4572-4942)	481.76 (464.19-499.93)	413 (297-547)	41.44 (29.84-54.88)	1023 (983-1065)	69.46 (66.78-72.2)	8795 (8445-9115)	568.84 (547.39-589.65)	747 (537-985)	48.77 (35.1-64.87)	0.45 (0.45-0.46)	0.54 (0.53-0.55)	0.53 (0.51-0.54)
Mexico	55532 (53580-57223)	72.95 (70.67-75.22)	417870 (404274-431896)	583.68 (564.95-603.15)	36566 (26418-48806)	50.22 (36.45-66.69)	104805 (101457-108044)	78.88 (76.32-81.32)	860983 (832430-888522)	643.31 (622.54-663.31)	74182 (54036-99119)	55.42 (40.39-73.99)	0.25 (0.24-0.26)	0.31 (0.31-0.31)	0.31 (0.3-0.32)
Micronesia (Federated States of)	42 (40-44)	50.36 (48.32-52.36)	286 (276-297)	369.69 (356.61-383.77)	25 (18-34)	31.82 (22.73-42.7)	56 (54-59)	57.03 (54.92-59.33)	410 (395-426)	425.26 (409.5-441.1)	36 (25-47)	36.58 (26.3-48.23)	0.4 (0.39-0.4)	0.44 (0.44-0.45)	0.44 (0.43-0.44)
Monaco	39 (38-41)	113.7 (109.78-117.79)	479 (463-496)	1196.08 (1152.63-1237.02)	41 (30-55)	103.92 (74.82-138.5)	54 (52-56)	124.07 (119.99-128.24)	656 (631-681)	1288.54 (1244.19-1336.33)	56 (41-74)	111.51 (81.52-148.48)	0.2 (0.16-0.24)	0.23 (0.23-0.24)	0.23 (0.22-0.24)
Mongolia	872 (834-909)	47.44 (45.62-49.33)	5701 (5472-5938)	333.78 (321.42-346.76)	499 (355-665)	28.85 (20.59-38.08)	1756 (1682-1827)	53.41 (51.18-55.62)	12281 (11830-12750)	387.74 (374.38-402.33)	1066 (773-1426)	33.47 (24.46-44.7)	0.41 (0.38-0.43)	0.49 (0.47-0.52)	0.49 (0.47-0.51)
Montenegro	358 (345-370)	55.4 (53.42-57.26)	2718 (2622-2818)	419.6 (405.16-434.44)	236 (170-315)	36.37 (26.27-48.7)	427 (411-443)	61.22 (59.16-63.45)	3559 (3431-3692)	476.24 (459.87-494.08)	304 (220-402)	41.21 (29.88-54.87)	0.39 (0.37-0.42)	0.49 (0.45-0.53)	0.48 (0.45-0.52)
Morocco	11429 (11001-11887)	51.55 (49.51-53.57)	79509 (76601-82558)	380.04 (365.24-394.35)	6904 (4924-9269)	32.65 (23.49-43.49)	23292 (22441-24291)	60.91 (58.72-63.44)	175290 (168441-182548)	459.05 (441.46-477.25)	15008 (10839-20036)	39.24 (28.44-52.32)	0.54 (0.54-0.55)	0.61 (0.59-0.62)	0.6 (0.58-0.61)
Mozambique	2265 (2175-2347)	20.22 (19.46-20.88)	13576 (13098-14094)	128.43 (124.07-132.74)	1191 (834-1603)	11.01 (7.79-14.74)	5361 (5140-5563)	20.93 (20.18-21.64)	31889 (30781-33073)	132.53 (128.4-136.92)	2811 (1988-3774)	11.32 (8.09-15)	0.15 (0.13-0.16)	0.14 (0.12-0.15)	0.14 (0.12-0.16)
Myanmar	18221 (17518-18961)	51.53 (49.61-53.53)	127950 (123099-132773)	380.19 (365.99-394.82)	11168 (8020-14907)	32.72 (23.55-43.54)	34762 (33340-36095)	61.59 (59.13-63.96)	265165 (254408-275934)	475.29 (456.04-493.22)	22985 (16524-30711)	40.99 (29.57-54.77)	0.62 (0.6-0.63)	0.78 (0.76-0.8)	0.8 (0.78-0.83)
Namibia	376 (361-391)	30.89 (29.61-	2517 (2413-2616)	220.49 (212.01-	221 (156-298)	19.09 (13.4-25.51)	764 (731-794)	33.53 (32.11-	5311 (5097-5533)	241.35 (232.09-	463 (334-639)	20.77 (15.07-	0.34 (0.31-	0.36 (0.33-	0.34 (0.31-

		32.02)		228.69)				34.75)		250.48)		28.16)	0.37)	0.39)	0.38)
Nauru	5 (4-5)	53.07 (51.01-55.19)	32 (30-33)	399.1 (384.87-415.06)	3 (2-4)	34.39 (24.61-45.57)	6 (6-6)	59.57 (57.35-61.9)	41 (40-43)	452.5 (435.94-470.74)	4 (3-5)	38.72 (27.76-52.37)	0.36 (0.3-0.42)	0.39 (0.31-0.47)	0.37 (0.29-0.45)
Nepal	6697 (6451-6939)	38.9 (37.64-40.28)	42571 (41088-44083)	262.47 (253.33-271.89)	3711 (2636-5044)	22.51 (16.08-30.25)	14421 (13902-14920)	47.55 (45.88-49.22)	101457 (97750-105363)	339.9 (327.89-352.4)	8815 (6439-11694)	29.31 (21.28-39.09)	0.61 (0.52-0.69)	0.76 (0.62-0.9)	0.78 (0.64-0.92)
Netherlands	17325 (16734-17954)	108.74 (105.12-112.32)	188620 (182524-195207)	1117.41 (1082.37-1156.07)	16283 (11707-21539)	97.02 (69.88-128.57)	23405 (22490-24249)	120.48 (115.97-124.54)	267514 (257151-277772)	1231.91 (1186.69-1280.52)	22795 (16554-29959)	106.59 (77.54-141.69)	0.24 (0.2-0.29)	0.3 (0.3-0.31)	0.3 (0.29-0.32)
New Zealand	2320 (2243-2399)	65.87 (63.66-68.09)	18552 (17916-19156)	515.4 (497.18-531.8)	1582 (1141-2117)	44.08 (31.83-58.85)	4247 (4094-4382)	74.27 (71.55-76.74)	34969 (33749-36098)	581.15 (561.07-602.02)	2977 (2158-3944)	50.04 (36.2-66.7)	0.29 (0.24-0.33)	0.36 (0.34-0.37)	0.37 (0.36-0.39)
Nicaragua	2213 (2111-2302)	66.92 (64.18-69.58)	15532 (14885-16180)	514.77 (495.44-534.65)	1367 (970-1850)	44.46 (31.68-59.47)	4796 (4603-4991)	73.99 (71.11-76.93)	37240 (35799-38665)	586.43 (564.59-608.63)	3238 (2331-4339)	50.6 (36.38-67.6)	0.31 (0.29-0.33)	0.41 (0.38-0.43)	0.41 (0.38-0.43)
Niger	1659 (1597-1717)	25.24 (24.34-26.09)	10016 (9635-10399)	165.19 (159.47-170.82)	886 (619-1219)	14.31 (9.97-19.51)	5242 (5039-5450)	25.83 (24.96-26.72)	31127 (29971-32256)	166.17 (160.66-171.4)	2776 (1979-3827)	14.49 (10.41-19.74)	0.2 (0.15-0.25)	0.15 (0.09-0.2)	0.17 (0.12-0.23)
Nigeria	38730 (37361-39858)	48.47 (46.95-49.95)	328727 (317592-339953)	453.05 (438.56-467.86)	28537 (20770-38216)	38.8 (28.14-51.55)	115537 (111284-119031)	56.29 (54.46-58.04)	1010164 (975218-1044865)	551.06 (533.66-569.53)	88465 (64176-118564)	47.43 (34.47-63.26)	0.59 (0.54-0.64)	0.74 (0.69-0.79)	0.77 (0.71-0.82)
Niue	1 (1-1)	52.18 (50.03-54.29)	8 (8-9)	389.23 (374.4-404.14)	1 (1-1)	33.54 (24.67-45.32)	1 (1-1)	60 (57.69-62.27)	9 (8-9)	459.21 (441.54-476.27)	1 (1-1)	39.54 (28.96-52.94)	0.48 (0.47-0.49)	0.57 (0.56-0.58)	0.56 (0.54-0.57)
North Macedonia	1098 (1060-1138)	53.6 (51.72-55.48)	8121 (7820-8405)	397.19 (382.91-410.7)	706 (504-941)	34.48 (24.62-45.88)	1519 (1460-1577)	60.32 (58.17-62.59)	12536 (12071-12990)	464.56 (448.35-480.6)	1069 (776-1431)	40.09 (29.14-54.18)	0.43 (0.41-0.46)	0.55 (0.53-0.57)	0.55 (0.53-0.58)
Northern Mariana Islands	25 (24-26)	59.04 (56.83-61.19)	179 (172-187)	462.83 (446.23-480.11)	16 (12-21)	40.26 (29.13-52.75)	34 (33-36)	63.32 (60.74-65.64)	273 (262-283)	496.77 (477.67-514.37)	24 (17-31)	42.97 (30.8-56.59)	0.19 (0.17-0.22)	0.17 (0.13-0.21)	0.16 (0.13-0.2)
Norway	3944 (3825-4072)	87.33 (84.66-90.2)	37935 (36749-39181)	784.28 (758.96-810.8)	3239 (2349-4323)	67.69 (49.04-90.38)	5684 (5501-5872)	94.04 (91.08-97.11)	54113 (52359-56016)	825.84 (799.72-854.46)	4628 (3339-6206)	71.53 (51.64-96.05)	0.16 (0.11-0.21)	0.17 (0.14-0.2)	0.19 (0.16-0.22)
Oman	925 (886-964)	55.27 (53.17-57.37)	6527 (6263-6778)	422.98 (407.02-438.82)	572 (414-790)	36.4 (26.23-49.54)	3334 (3193-3501)	69.93 (67.41-72.65)	26352 (25162-27590)	568.97 (547.4-590.68)	2309 (1632-3124)	49.1 (35.04-65.78)	0.79 (0.75-0.83)	1 (0.95-1.05)	1.01 (0.95-1.06)

Pakistan	40870 (39330-42220)	41.92 (40.58-43.32)	268780 (259688-277905)	294.11 (284.29-303.76)	23502 (16561-31350)	25.37 (18.05-33.5)	103854 (100121-107328)	47.29 (45.7-48.83)	706459 (681703-732164)	336.42 (325.37-347.8)	61673 (44495-82447)	28.98 (21.11-38.52)	0.39 (0.39-0.4)	0.44 (0.43-0.45)	0.44 (0.43-0.45)
Palau	8 (8-8)	55.96 (53.75-58.13)	58 (56-61)	431.25 (414.64-448.74)	5 (4-7)	37.08 (26.65-49.87)	14 (13-14)	63.19 (60.86-65.68)	109 (105-114)	490.77 (471.37-509.82)	9 (7-13)	42.15 (30.52-56.37)	0.37 (0.35-0.39)	0.4 (0.38-0.42)	0.38 (0.36-0.4)
Palestine	812 (782-844)	50.37 (48.61-52.32)	5382 (5187-5581)	366.7 (353.51-380.14)	470 (332-636)	31.51 (22.63-42.29)	2845 (2739-2958)	61.16 (58.85-63.47)	20252 (19453-21050)	462.36 (444.45-481.55)	1750 (1258-2346)	39.42 (28.56-53.24)	0.65 (0.64-0.65)	0.77 (0.75-0.79)	0.75 (0.73-0.77)
Panama	1686 (1615-1751)	76.29 (73.25-79.31)	13386 (12886-13913)	631.98 (608.72-654.97)	1174 (833-1548)	54.85 (39.13-72.33)	3577 (3432-3713)	82.33 (79-85.51)	30400 (29217-31472)	695.77 (668.82-720.11)	2632 (1916-3489)	60.28 (43.83-79.84)	0.24 (0.22-0.25)	0.3 (0.28-0.31)	0.29 (0.27-0.3)
Papua New Guinea	1546 (1481-1608)	45.87 (44.1-47.7)	10158 (9738-10545)	322.91 (309.59-334.85)	884 (630-1162)	27.58 (19.8-36.17)	4704 (4500-4892)	51.55 (49.35-53.58)	31615 (30415-32822)	368.33 (354.74-382.64)	2773 (1947-3697)	31.78 (22.67-41.7)	0.36 (0.35-0.37)	0.4 (0.39-0.42)	0.42 (0.41-0.43)
Paraguay	2023 (1947-2090)	56.19 (54.24-58.02)	13718 (13242-14198)	402.18 (388.01-415.97)	1204 (868-1615)	34.85 (25.16-46.34)	4084 (3946-4226)	57.96 (55.96-59.91)	28926 (27963-30042)	413.76 (399.91-428.86)	2506 (1796-3330)	35.69 (25.57-47.44)	0.09 (0.07-0.1)	0.07 (0.06-0.08)	0.06 (0.05-0.07)
Peru	18350 (17653-19029)	92.75 (89.25-96.04)	160362 (154311-166194)	864.92 (832.8-895.82)	14067 (10170-19026)	75.07 (54.05-100.53)	38530 (37066-39994)	105.83 (101.84-109.77)	374463 (360745-388837)	1029.54 (992.9-1067.86)	32590 (23619-43636)	89.47 (64.86-119.72)	0.45 (0.42-0.49)	0.6 (0.55-0.65)	0.61 (0.55-0.67)
Philippines	31641 (30606-32603)	59.62 (57.85-61.46)	231768 (223825-239191)	469.06 (453.6-483.82)	20283 (14572-27014)	40.36 (29.16-53.76)	72611 (70393-74978)	66.84 (64.79-68.9)	562035 (543414-580355)	532.32 (514.98-549.02)	48891 (35465-65626)	45.91 (33.32-61.38)	0.35 (0.33-0.37)	0.38 (0.36-0.41)	0.4 (0.38-0.42)
Poland	23518 (22780-24318)	58.59 (56.78-60.54)	186184 (179873-192446)	455.81 (440.76-470.54)	15921 (11474-21145)	39.13 (28.21-51.98)	27814 (27097-28555)	63.91 (62.44-65.51)	243480 (239071-247325)	509.08 (500.31-517.12)	20640 (15172-27290)	43.9 (32.1-58.24)	0.3 (0.28-0.32)	0.36 (0.33-0.38)	0.38 (0.36-0.41)
Portugal	10964 (10574-11358)	100.73 (97.2-104.24)	113670 (109844-117874)	993.24 (959.47-1029.15)	9703 (6953-13021)	85.34 (60.91-114.4)	13895 (13400-14413)	114.65 (110.8-118.68)	158343 (152596-164258)	1142.43 (1100.97-1184.51)	13402 (9649-17638)	98.47 (70.38-130.18)	0.33 (0.28-0.37)	0.44 (0.42-0.45)	0.45 (0.43-0.47)
Puerto Rico	2881 (2764-2995)	79.94 (76.7-83.07)	24660 (23683-25602)	678.45 (652.36-704.27)	2141 (1545-2851)	58.89 (42.55-78.3)	3179 (3043-3311)	86.63 (83.22-89.97)	30771 (29548-31888)	752.34 (724.74-778.23)	2611 (1892-3439)	65.15 (47.41-86.92)	0.27 (0.25-0.28)	0.34 (0.33-0.35)	0.34 (0.32-0.35)
Qatar	257 (244-269)	59.69 (57.41-61.97)	1939 (1843-2035)	476.92 (459.35-494.68)	171 (122-232)	41.21 (29.75-55.48)	2345 (2224-2473)	71.84 (68.94-74.61)	19125 (18115-20079)	592.5 (569.75-616.12)	1681 (1168-2294)	51.06 (36.46-68.99)	0.58 (0.55-0.61)	0.66 (0.63-0.7)	0.66 (0.62-0.7)
Republic of Korea	21864	50.29	149821	350.37	13087	30.34	36302	57.19	268360	399.58	23037	34.83	0.3 (0.25-	0.39	0.4 (0.36-

	(20959-22742)	(48.32-52.17)	(144007-155519)	(337.46-362.98)	(9204-17601)	(21.45-40.48)	(34883-37742)	(55.13-59.29)	(258317-278480)	(384.99-414)	(16603-30580)	(25.05-46.06)	0.35)	(0.35-0.42)	0.44)
Republic of Moldova	2301 (2209-2398)	50.52 (48.52-52.64)	16619 (15999-17282)	364.94 (351.03-379.2)	1430 (1035-1940)	31.42 (22.69-42.64)	2360 (2253-2466)	56.57 (54.24-58.94)	18872 (18037-19600)	421.29 (403.89-437.69)	1608 (1134-2160)	36.36 (25.69-48.56)	0.4 (0.38-0.43)	0.49 (0.46-0.53)	0.51 (0.48-0.55)
Romania	13290 (12809-13793)	52.93 (51.1-54.85)	99981 (96133-103911)	389.33 (374.78-404.85)	8581 (6262-11323)	33.57 (24.42-44.68)	12665 (12229-13136)	58.37 (56.25-60.3)	104457 (100751-108522)	440.15 (424.6-455.79)	8911 (6434-11850)	38.17 (27.63-51.08)	0.36 (0.34-0.39)	0.43 (0.41-0.46)	0.46 (0.43-0.48)
Russian Federation	85725 (82967-88625)	52.95 (51.32-54.7)	645876 (624145-667266)	389.58 (376.03-401.93)	55129 (40054-73275)	33.43 (24.28-44.52)	97499 (94380-100750)	58.94 (57.1-60.93)	788018 (760583-814991)	445.64 (430.81-459.64)	66648 (48786-88847)	38.24 (27.71-50.87)	0.37 (0.35-0.39)	0.45 (0.43-0.47)	0.46 (0.44-0.49)
Rwanda	1066 (1013-1114)	18.22 (17.41-19.08)	6170 (5891-6441)	112.78 (108.12-117.86)	550 (383-725)	9.81 (6.94-12.96)	2225 (2123-2326)	18.92 (18.11-19.74)	13190 (12591-13810)	116.99 (111.34-122.08)	1168 (823-1600)	10.17 (7.17-13.85)	0.18 (0.16-0.21)	0.18 (0.16-0.21)	0.19 (0.15-0.22)
Saint Kitts and Nevis	30 (28-31)	76.37 (73.22-79.43)	242 (233-251)	632.14 (608.7-656.39)	21 (15-28)	54.58 (39.18-73.42)	54 (52-57)	83.25 (79.89-86.48)	477 (457-497)	706.93 (678.65-733.46)	41 (29-54)	60.87 (43.46-81.21)	0.27 (0.26-0.29)	0.36 (0.34-0.37)	0.36 (0.34-0.37)
Saint Lucia	90 (86-94)	72.6 (69.43-75.46)	690 (665-718)	583.74 (562.4-608.02)	60 (43-80)	50.24 (36-66.6)	157 (150-164)	79.84 (76.68-83.23)	1356 (1302-1414)	661.37 (636.62-687.54)	116 (84-153)	56.86 (41.26-76.01)	0.3 (0.27-0.32)	0.39 (0.37-0.41)	0.39 (0.37-0.42)
Saint Vincent and the Grenadines	71 (68-74)	71.56 (68.63-74.48)	544 (522-564)	570.8 (549.94-592.61)	48 (34-63)	49.47 (35.79-64.89)	95 (92-99)	78.07 (74.98-81.22)	805 (774-837)	638.44 (615.01-663.04)	69 (50-91)	54.95 (39.79-72.46)	0.27 (0.26-0.29)	0.35 (0.33-0.37)	0.34 (0.33-0.36)
Samoa	71 (68-74)	51.79 (49.8-53.89)	497 (479-516)	386.09 (371.76-400.22)	44 (31-58)	33.34 (23.92-44.38)	111 (107-115)	58.16 (55.8-60.28)	806 (777-836)	438.2 (422.48-454.68)	70 (51-94)	37.7 (27.28-50.24)	0.38 (0.37-0.38)	0.42 (0.41-0.42)	0.41 (0.4-0.42)
San Marino	29 (28-30)	111.95 (107.98-115.8)	321 (310-334)	1169.15 (1128.48-1213.67)	28 (20-37)	101.43 (72.58-135.29)	46 (44-48)	122 (117.72-126.38)	536 (516-556)	1257.83 (1213.27-1301.05)	45 (33-60)	108.56 (78.45-144.19)	0.2 (0.16-0.24)	0.23 (0.21-0.25)	0.22 (0.2-0.24)
Sao Tome and Principe	31 (29-32)	29.37 (28.39-30.39)	202 (195-209)	207.46 (200.25-214.37)	18 (13-24)	18.02 (12.64-24.54)	65 (62-67)	32.23 (31.14-33.28)	444 (429-460)	230.48 (222.9-238.45)	39 (27-53)	20.02 (14.26-27.07)	0.46 (0.4-0.52)	0.52 (0.46-0.59)	0.53 (0.46-0.61)
Saudi Arabia	7570 (7254-7856)	56.62 (54.45-58.75)	53964 (51801-56061)	440.67 (423.7-457.4)	4732 (3369-6456)	37.9 (26.97-51.05)	28543 (27269-29852)	70.82 (68.27-73.42)	231869 (221495-242682)	582.96 (561.04-605.15)	20085 (14133-27171)	49.87 (35.13-67.38)	0.76 (0.74-0.79)	0.96 (0.93-0.98)	0.94 (0.91-0.97)
Senegal	1773 (1705-	27.67 (26.72-	11227 (10816-	189.04 (182-	985 (698-1297)	16.28 (11.78-	4242 (4079-	30.05 (29-31.01)	27754 (26746-	206.68 (199.76-	2437 (1776-	17.89 (13.18-	0.38 (0.34-	0.4 (0.36-0.45)	0.42 (0.37-

	1836)	28.62)	11653)	195.95)		21.25)	4381)		28878)	213.94)	3304)	24.08)	0.43)		0.47)
Serbia	5712 (5494-5923)	54.08 (51.96-55.95)	43488 (41895-45215)	402.59 (387.91-418.3)	3756 (2664-4973)	34.93 (24.66-46.51)	6198 (5985-6427)	60.93 (58.83-63.05)	52586 (50731-54563)	472.54 (455.56-489.9)	4491 (3208-5919)	41.05 (29.12-54.09)	0.44 (0.42-0.47)	0.58 (0.55-0.6)	0.58 (0.55-0.61)
Seychelles	43 (41-44)	64.12 (61.51-66.52)	340 (327-352)	524.09 (505.01-544.17)	30 (21-39)	45.5 (32.22-60.02)	84 (81-88)	72.7 (69.73-75.68)	711 (681-739)	605.83 (582.69-628.91)	61 (44-82)	52.26 (38.01-69.97)	0.38 (0.36-0.4)	0.44 (0.41-0.46)	0.42 (0.39-0.45)
Sierra Leone	990 (950-1026)	27.71 (26.75-28.71)	6365 (6143-6602)	189.37 (182.97-195.46)	555 (388-753)	16.3 (11.48-21.8)	2292 (2212-2372)	29.43 (28.44-30.41)	14760 (14226-15327)	200.2 (193.59-206.91)	1300 (915-1750)	17.34 (12.37-23.27)	0.34 (0.28-0.4)	0.33 (0.26-0.4)	0.36 (0.28-0.44)
Singapore	1668 (1602-1739)	54.26 (52.3-56.39)	12027 (11545-12491)	392.43 (378.28-406.37)	1056 (752-1420)	34.21 (24.54-45.58)	4178 (4006-4337)	61.19 (58.82-63.44)	31631 (30398-32813)	442.05 (426.57-458.39)	2751 (1954-3649)	38.77 (27.4-51.29)	0.3 (0.25-0.36)	0.4 (0.36-0.43)	0.41 (0.37-0.44)
Slovakia	2977 (2872-3077)	54.1 (52.16-55.94)	22594 (21814-23391)	403.47 (389.66-417.72)	1943 (1406-2543)	34.79 (25.25-45.81)	3794 (3648-3927)	60.97 (58.82-63.01)	32001 (30815-33264)	473.37 (456.69-491.63)	2728 (1986-3614)	40.99 (29.54-54.62)	0.42 (0.39-0.45)	0.53 (0.49-0.57)	0.56 (0.52-0.59)
Slovenia	1198 (1158-1241)	56.2 (54.25-58.2)	9494 (9148-9858)	430.97 (415.37-447.08)	813 (596-1095)	37.12 (27.19-50.09)	1493 (1436-1548)	62.68 (60.38-64.91)	13055 (12504-13536)	494.64 (476.09-513.71)	1104 (796-1476)	42.68 (31.05-57.09)	0.38 (0.35-0.41)	0.46 (0.43-0.48)	0.47 (0.44-0.5)
Solomon Islands	119 (114-124)	44.98 (43.16-46.83)	773 (742-802)	315.01 (302.87-326.38)	68 (48-94)	27.17 (19.66-36.99)	304 (292-317)	51.12 (49.2-53.18)	2038 (1958-2115)	362.79 (349.45-375.93)	178 (126-241)	31.25 (22.4-42.06)	0.4 (0.38-0.41)	0.43 (0.41-0.45)	0.43 (0.41-0.45)
Somalia	1262 (1210-1307)	19.71 (19-20.35)	7402 (7143-7680)	124.36 (120.61-128.4)	659 (475-877)	10.76 (7.69-14.22)	3215 (3084-3340)	18.57 (17.93-19.21)	18561 (17877-19287)	114.89 (111.12-118.48)	1664 (1165-2277)	9.99 (7.07-13.5)	-0.17 (-0.18--0.17)	-0.25 (-0.25--0.24)	-0.22 (-0.24--0.21)
South Africa	10940 (10546-11306)	32.51 (31.42-33.56)	75792 (73154-78435)	235.87 (228.12-243.69)	6594 (4723-8900)	20.26 (14.65-27.21)	19797 (19146-20459)	34.53 (33.42-35.63)	142746 (138100-147794)	250.56 (242.59-259.21)	12171 (8728-16250)	21.28 (15.3-28.48)	0.28 (0.25-0.31)	0.28 (0.25-0.31)	0.25 (0.21-0.28)
South Sudan	1052 (1011-1090)	21.57 (20.78-22.26)	6445 (6214-6688)	140.04 (135.32-144.27)	567 (406-762)	12.01 (8.61-15.97)	1704 (1637-1767)	20.95 (20.22-21.66)	10224 (9854-10605)	132.67 (128.41-137.2)	903 (641-1218)	11.4 (8.05-15.33)	-0.06 (-0.08--0.04)	-0.14 (-0.16--0.11)	-0.13 (-0.15--0.1)
Spain	32879 (31713-34047)	78.91 (76.18-81.72)	292923 (282611-303253)	672.28 (648.54-697.6)	25246 (18203-33583)	58.34 (41.91-77.74)	45678 (44062-47212)	86.74 (84.04-89.49)	426379 (415499-439628)	734 (715.17-756.45)	36353 (26639-48530)	63.59 (46.19-84.17)	0.17 (0.1-0.23)	0.19 (0.14-0.24)	0.19 (0.14-0.24)
Sri Lanka	9735 (9339-10126)	61.3 (58.95-63.7)	74870 (71851-77763)	492.51 (473.1-510.15)	6526 (4713-8719)	42.44 (30.73-56.11)	17118 (16422-17856)	70.79 (67.85-73.68)	145081 (139392-150999)	585.81 (563.84-609.46)	12404 (9081-16247)	50.32 (36.96-66.24)	0.47 (0.46-0.48)	0.56 (0.55-0.58)	0.56 (0.54-0.57)

Sudan	8132 (7817-8441)	49.66 (47.89-51.53)	54539 (52489-56446)	358.84 (345.27-372.18)	4772 (3360-6604)	30.92 (22.03-42.36)	23667 (22674-24593)	61.85 (59.5-64.02)	168075 (161916-174218)	470.67 (453.34-487.81)	14635 (10465-19875)	40.39 (28.89-54.55)	0.74 (0.73-0.75)	0.92 (0.9-0.94)	0.93 (0.9-0.95)
Suriname	273 (261-284)	75.15 (72.08-78.05)	2153 (2071-2234)	616.13 (593.13-639.35)	188 (134-255)	53.34 (38.39-72.62)	487 (467-507)	80.28 (77.08-83.53)	4101 (3939-4251)	667.08 (641.53-691.16)	351 (256-469)	57.3 (41.67-76.53)	0.24 (0.23-0.25)	0.29 (0.28-0.31)	0.28 (0.27-0.3)
Sweden	8236 (7960-8502)	88.65 (85.62-91.58)	81030 (78061-83817)	803 (775.34-830.44)	6932 (4980-9160)	69.56 (49.8-92.18)	10761 (10408-11125)	94.33 (91.3-97.36)	104275 (100752-108242)	830.15 (802.7-860.51)	8898 (6524-11964)	71.94 (52.76-97.96)	0.09 (0.04-0.15)	0.06 (0.04-0.09)	0.06 (0.03-0.09)
Switzerland	8509 (8216-8809)	114.36 (110.35-118.33)	96791 (93317-100238)	1204.33 (1163.62-1247.09)	8267 (5973-10932)	103.71 (75.26-137.1)	12563 (12118-13012)	124.84 (120.72-129.1)	146598 (140949-152012)	1299.06 (1250.27-1345.1)	12461 (9005-16677)	112.12 (80.99-149.88)	0.21 (0.17-0.25)	0.25 (0.24-0.25)	0.26 (0.26-0.26)
Syrian Arab Republic	5253 (5055-5424)	51.58 (49.75-53.39)	35357 (34074-36723)	380.39 (366.43-394.37)	3098 (2199-4173)	32.81 (23.58-43.67)	9112 (8716-9486)	62.84 (60.38-65.21)	70002 (67257-72613)	484.19 (465.69-501.55)	5987 (4280-8026)	41.34 (29.67-55.6)	0.68 (0.64-0.71)	0.83 (0.79-0.86)	0.81 (0.77-0.85)
Taiwan (Province of China)	6382 (6153-6627)	32.08 (30.95-33.24)	39634 (38489-41035)	202.69 (196.85-209.87)	3501 (2507-4725)	17.79 (12.66-23.91)	11951 (11511-12346)	40.14 (38.79-41.4)	81535 (79276-83931)	260.1 (253.19-267.38)	7009 (5112-9332)	22.67 (16.53-30.06)	0.87 (0.79-0.95)	0.99 (0.88-1.1)	0.97 (0.87-1.08)
Tajikistan	2213 (2125-2311)	48.91 (47.05-51.01)	14616 (14065-15225)	349.18 (335.97-362.45)	1287 (922-1721)	30.38 (21.95-40.47)	4999 (4804-5187)	52.72 (50.69-54.75)	34254 (32884-35665)	380.04 (365.13-394.35)	2988 (2137-3990)	32.86 (23.47-44.06)	0.29 (0.25-0.33)	0.31 (0.25-0.38)	0.31 (0.24-0.37)
Thailand	31377 (30027-32613)	59.35 (56.99-61.62)	239706 (230514-248579)	469.9 (452.55-486.3)	20945 (15045-28049)	40.58 (29.26-53.89)	54851 (52544-57098)	68.35 (65.64-70.97)	476545 (458133-494877)	556.82 (536.16-577.95)	40794 (29663-54611)	48.21 (34.69-64.64)	0.45 (0.44-0.46)	0.54 (0.52-0.55)	0.55 (0.53-0.56)
Timor-Leste	331 (316-345)	52.55 (50.43-54.76)	2261 (2170-2352)	392.06 (376.65-407.16)	198 (140-268)	33.62 (24.38-45.14)	770 (737-800)	63.42 (60.78-65.98)	5759 (5542-5968)	496.78 (478-513.99)	502 (366-677)	42.75 (31.23-57.41)	0.67 (0.65-0.7)	0.85 (0.82-0.89)	0.88 (0.84-0.92)
Togo	863 (828-894)	28.46 (27.41-29.44)	5497 (5282-5708)	197.35 (190.47-204.12)	483 (345-638)	17.01 (12.15-22.69)	2301 (2216-2389)	30.43 (29.42-31.48)	15125 (14584-15688)	210.57 (203.23-217.63)	1331 (959-1826)	18.28 (13.22-24.85)	0.33 (0.29-0.38)	0.32 (0.28-0.37)	0.33 (0.29-0.38)
Tokelau	1 (1-1)	49.21 (47.16-51.05)	5 (5-5)	359.75 (345.29-373.22)	0 (0-1)	31.12 (22.14-42.1)	1 (1-1)	58.69 (56.35-60.94)	6 (6-7)	444.09 (427.33-460.7)	1 (0-1)	38.12 (27.81-50.57)	0.59 (0.58-0.6)	0.71 (0.69-0.72)	0.68 (0.66-0.7)
Tonga	41 (40-43)	50.08 (48.19-52.14)	287 (277-297)	369.06 (355.64-382.35)	25 (18-34)	32.03 (22.39-42.69)	55 (53-57)	57.07 (54.88-59.39)	401 (385-415)	427.76 (410.91-443.38)	35 (25-47)	36.83 (26.07-49.55)	0.41 (0.4-0.41)	0.46 (0.45-0.47)	0.44 (0.43-0.45)
Trinidad and	905 (868-	79.21	7369 (7088-	668.99	642 (461-	57.83	1302	85.39	11758	735.06	1002 (711-	63.21	0.27	0.35	0.34

Tobago	941)	(76.11-82.41)	7656)	(643.73-696.79)	852)	(41.64-76.74)	(1246-1357)	(81.8-88.98)	(11304-12223)	(707.97-762.23)	1327)	(44.7-83.43)	(0.26-0.29)	(0.33-0.37)	(0.32-0.36)
Tunisia	4005 (3861-4157)	53.64 (51.79-55.66)	28627 (27597-29702)	404.8 (390.26-420.41)	2492 (1828-3364)	34.9 (25.56-47.07)	8214 (7872-8541)	64.52 (61.8-66.94)	65527 (63049-68234)	503.58 (484.85-523.13)	5593 (3985-7505)	43.11 (30.73-57.73)	0.61 (0.58-0.63)	0.71 (0.68-0.75)	0.7 (0.66-0.74)
Turkmenistan	1649 (1582-1715)	51.46 (49.58-53.42)	11220 (10770-11679)	376.14 (361.8-390.49)	985 (698-1329)	32.63 (23.12-43.71)	2922 (2807-3043)	56.81 (54.59-59.2)	21380 (20570-22186)	424.95 (409.09-441.19)	1868 (1368-2504)	36.97 (27.08-49.49)	0.38 (0.35-0.4)	0.44 (0.4-0.48)	0.45 (0.41-0.49)
Tuvalu	4 (4-4)	48.88 (46.95-50.83)	30 (28-31)	356.82 (343.4-370.37)	3 (2-3)	30.83 (22.24-40.87)	7 (7-7)	57.5 (55.3-59.73)	50 (48-52)	428.44 (412.22-444.35)	4 (3-6)	36.98 (26.42-49.85)	0.51 (0.5-0.52)	0.57 (0.56-0.58)	0.58 (0.57-0.59)
Turkiye	33716 (32438-34938)	64.31 (62.03-66.67)	267838 (257300-277914)	541.47 (520.76-561.82)	23253 (16415-30882)	46.59 (33.14-61.95)	71700 (69085-74506)	79.72 (76.79-82.8)	649200 (623744-674172)	704.88 (677.76-731.56)	55689 (40383-74010)	60.61 (43.95-80.78)	0.74 (0.72-0.76)	0.92 (0.89-0.94)	0.91 (0.89-0.94)
Uganda	2940 (2823-3053)	21.32 (20.53-22.1)	17659 (16986-18305)	137.73 (133.25-142.33)	1558 (1092-2115)	11.85 (8.49-15.97)	8040 (7725-8338)	22.73 (21.85-23.45)	48895 (47230-50711)	147.83 (142.86-152.58)	4345 (3065-5977)	12.78 (9.17-17.33)	0.27 (0.25-0.29)	0.29 (0.27-0.32)	0.32 (0.29-0.35)
Ukraine	30678 (29551-31808)	53.18 (51.32-55.01)	234151 (225234-242722)	390.77 (376.51-404.5)	19924 (14458-26647)	33.54 (24.43-44.76)	29042 (28089-30025)	57.7 (55.96-59.7)	234954 (226407-243278)	431.12 (415.61-446.51)	19797 (14461-26416)	36.92 (26.63-49.39)	0.3 (0.28-0.33)	0.35 (0.33-0.38)	0.36 (0.33-0.39)
United Arab Emirates	1053 (1010-1106)	60.22 (58.04-62.39)	7925 (7563-8306)	483.95 (467.16-502.36)	698 (495-945)	41.76 (30.25-56)	8286 (7804-8830)	72.78 (69.97-75.96)	71137 (67098-75082)	606.63 (583.52-629.93)	6195 (4347-8376)	52.29 (37.49-69.46)	0.61 (0.56-0.65)	0.7 (0.65-0.76)	0.7 (0.65-0.75)
United Kingdom	58227 (56537-60077)	94.97 (92.11-98)	596932 (578999-615094)	908.8 (880.64-936.68)	51143 (37166-68564)	78.58 (56.76-105.33)	81326 (78879-83987)	108.07 (104.75-111.46)	867723 (839922-895505)	1046.71 (1012.96-1080.29)	73776 (53401-98325)	90.22 (65.29-120.56)	0.3 (0.24-0.36)	0.39 (0.33-0.44)	0.38 (0.32-0.44)
United Republic of Tanzania	4825 (4574-5063)	22.77 (21.68-23.82)	29914 (28186-31580)	150.93 (142.64-158.9)	2626 (1834-3462)	12.95 (9.07-17.09)	11790 (11218-12334)	23.55 (22.5-24.65)	73731 (69784-77826)	155.44 (147.4-164.28)	6474 (4630-8870)	13.38 (9.47-18.09)	0.13 (0.12-0.14)	0.11 (0.1-0.12)	0.15 (0.13-0.16)
United States of America	254425 (247149-262221)	97.44 (94.56-100.44)	2579771 (2503078-2662181)	946.53 (918.67-975.13)	220664 (160362-294717)	81.44 (58.99-108.69)	380893 (370221-391421)	103.97 (101.4-106.57)	3906305 (3827071-3984120)	983.5 (965.15-1003.32)	326749 (239298-431398)	83.61 (60.92-110.71)	0.1 (0.05-0.14)	0.13 (0.11-0.15)	0.1 (0.08-0.12)
United States Virgin Islands	85 (82-89)	80.71 (77.56-83.86)	716 (689-742)	688.89 (664.14-714.93)	62 (45-83)	59.87 (43.41-79.15)	85 (81-89)	86.29 (82.78-89.69)	817 (785-850)	747.96 (720.15-775.83)	69 (50-91)	64.46 (46.53-85.85)	0.22 (0.2-0.23)	0.26 (0.25-0.28)	0.25 (0.23-0.27)
Uruguay	2205 (2127-	67.87 (65.42-	17797 (17130-	537.21 (517.01-	1535 (1117-	46.54 (33.72-	2810 (2699-	75.87 (72.79-	23347 (22482-	597.9 (575.63-	1991 (1424-	51.53 (37.05-	0.26 (0.22-	0.34 (0.33-	0.33 (0.33-

	2284)	70.36)	18437)	556.76)	2019)	61.38)	2921)	78.68)	24205)	620.15)	2641)	68.79)	0.31)	0.35)	0.34)
Uzbekistan	8887 (8521-9232)	49.17 (47.33-51.2)	59452 (57158-61928)	351.87 (338.91-365.93)	5199 (3756-6903)	30.43 (22-40.14)	18926 (18193-19705)	55.39 (53.22-57.7)	136910 (131440-142234)	409.03 (392.79-423.95)	11909 (8470-15748)	35.43 (25.18-46.74)	0.43 (0.41-0.44)	0.52 (0.5-0.53)	0.52 (0.5-0.54)
Vanuatu	56 (54-59)	46.58 (44.96-48.4)	370 (355-384)	330.59 (317.87-342.8)	32 (23-44)	28.5 (20.32-38.12)	145 (139-151)	52.4 (50.41-54.61)	989 (952-1026)	376.45 (362.49-391.53)	87 (62-116)	32.51 (23.59-43.43)	0.39 (0.38-0.4)	0.43 (0.42-0.44)	0.43 (0.42-0.44)
Venezuela (Bolivarian Republic of)	12757 (12206-13284)	75.16 (72.12-78.27)	98768 (94861-102703)	617.39 (594.85-640.33)	8673 (6187-11559)	53.54 (38.03-70.9)	22139 (21224-23008)	78.89 (75.42-81.98)	185494 (178715-192739)	650.05 (627.09-674.5)	15998 (11724-21333)	56.24 (41.07-75.11)	0.16 (0.13-0.19)	0.17 (0.14-0.2)	0.17 (0.13-0.2)
Viet Nam	32266 (31027-33579)	55.52 (53.42-57.74)	234511 (225654-243683)	425.93 (410.69-442.5)	20536 (14939-27873)	36.89 (26.71-49.55)	70452 (67528-73477)	66.3 (63.67-69.03)	561517 (537641-585401)	529.76 (508.25-551.39)	49017 (35650-65922)	46.16 (33.63-62.09)	0.59 (0.59-0.6)	0.73 (0.72-0.74)	0.75 (0.74-0.76)
Yemen	5036 (4836-5215)	48.43 (46.63-50.12)	32673 (31380-33936)	345.34 (332.62-358.17)	2850 (2040-3794)	29.52 (21.05-39.15)	17119 (16461-17791)	59.12 (56.7-61.54)	118284 (113680-122847)	438.14 (421.77-454.73)	10260 (7459-13745)	37.43 (27.34-49.85)	0.69 (0.66-0.72)	0.84 (0.81-0.87)	0.84 (0.8-0.87)
Zambia	1429 (1370-1484)	22.35 (21.56-23.13)	8736 (8407-9064)	146.86 (142.22-152.16)	772 (527-1035)	12.67 (8.77-16.87)	3832 (3676-3969)	23.26 (22.42-24.05)	23690 (22812-24591)	152.7 (147.26-157.72)	2093 (1502-2858)	13.16 (9.5-17.57)	0.18 (0.15-0.22)	0.18 (0.15-0.22)	0.18 (0.14-0.23)
Zimbabwe	2631 (2509-2745)	30.15 (28.99-31.35)	17223 (16540-17897)	213.49 (205.86-221.16)	1515 (1047-2011)	18.43 (13.06-24.12)	4310 (4126-4478)	31.06 (29.72-32.18)	28513 (27408-29768)	217.12 (209.23-225.33)	2497 (1759-3353)	18.69 (13.32-24.75)	0.1 (0.08-0.12)	0.03 (0-0.05)	0.02 (-0.01-0.05)

No., number; DALYs, disability-adjusted-life-years; ASIR, age-standardized incidence rate; ASPR, age-standardized prevalence rate; ASDR, age-standardized DALY rate; UI: uncertainty intervals; CI: confidence interval; SDI: socio-demographic index; GBD: global burden of diseases; EAPC: estimated annual percentage change.
